# Mechanisms of gut microbiota in host fat deposition: metabolites, signaling pathways, and translational applications

**DOI:** 10.3389/fmicb.2025.1675155

**Published:** 2025-12-18

**Authors:** Sha Liu

**Affiliations:** National Key Laboratory for Pig Genetic Improvement and Germplasm Innovation, Jiangxi Agricultural University, Nanchang, China

**Keywords:** gut microbiota, fat deposition, metabolites, epigenetics, signaling pathways, probiotics and prebiotics

## Abstract

Obesity and metabolic diseases are global health challenges, with gut microbiota playing a critical role in host fat deposition through symbiotic interactions. In recent years, the gut microbiota, as an important factor regulating fat deposition, has received widespread attention. Numerous studies have confirmed that gut microbes influence host fat accumulation by regulating energy metabolism, inflammatory response, and gut barrier function. In this review, we summarized the key roles of gut microbial metabolites, including short-chain fatty acids (SCFAs), bile acids, tryptophan metabolites, lipopolysaccharides (LPS), branched-chain amino acids (BCAAs), and trimethylamine N-oxide (TMAO) in host epigenetic regulation and lipid metabolism, and explored their regulatory mechanisms through mediated signaling pathways, including Wnt/*β*-catenin signaling pathway, transforming growth factor beta/SMAD3 pathway (TGF-*β*/SMAD3), peroxisome proliferator-activated receptor gamma (PPARγ), CCAAT/enhancer-binding protein alpha (C/EBPα), and nuclear factor kappa-light-chain-enhancer of activated B cells (NF-κB). In terms of translational applications, we described the research progress and application potentials of intervention strategies, such as probiotics, prebiotics, synbiotics, postbiotics, and fecal transplantation in obesity control and animal production. Finally, we proposed the current bottlenecks and translational challenges in obesity control by precision nutrition and microecological intervention, and look forward to future directions. This review provides a theoretical basis for the in-depth understanding of the interactions between gut microbiota and host metabolism, and serves as a reference for the prevention and control of metabolic diseases by developing nutritional intervention strategies for animals.

## 1 Introduction

Metabolic diseases, such as obesity, have become a major threaten to human health in the 21st century. According to the report, between 1990 and 2021, the rates of overweight and obesity in all over the world, have increased. Compared to 1990, the prevalence of obesity among men globally has risen by 155.1%, and among women it has increased by 104.9%. Under this trend, the total number of overweight and obese adults worldwide will reach 3.8 billion by 2050, which will exceed half of the global adult population (Collaborators, 2025). Obesity not only significantly increases the risk of complications such as type 2 diabetes and cardiovascular diseases, but also imposes a substantial burden on the public health system (Lyu et al., 2021). Furthermore, understanding the regulatory mechanisms of fat deposition is relevant to different species. For instance, in animal production, excessive fat accumulation lowers production efficiency and leads to economic losses (Chen et al., 2021). Therefore, investigating the mechanisms governing fat deposition is of significant theoretical importance and has broad implications across species.

Traditionally, obesity is considered a multifactorial disease, which is influenced by the complex interaction of genetics, environmental factors (especially diet and lifestyle), and gut microbiota (Młynarska et al., 2025). While the host’s genetic background determines metabolic susceptibility, the rapid increase in the prevalence of global obesity demonstrates that environmental factors, especially those capable of altering the gut microecology, also play a crucial role (Zsálig et al., 2023). As a dynamic bridge between the host and the environment, the gut microbiota is profoundly influenced by diet, utilization of antibiotics, and other lifestyle factors (Ramasinghe et al., 2025). At the same time, the gut microbiota also plays a key regulatory role by translating the influence of environmental and genetic factors into the metabolic characteristics of the host. The studies in humans and animals indicate that gut microbes can regulate the host’s energy metabolism, inflammatory responses, and lipogenesis, thereby influencing fat deposition (Takeuchi et al., 2023; Lai et al., 2024). The early studies have already discovered that transplanting the gut microbiota of obese mice with hereditary leptin deficiency (*ob/ob*) into germ-free (GF) mice could significantly increase the body weight and fat content of the recipient mice (Turnbaugh et al., 2006). This shows that even obesity phenotypes caused by genetic factors can be manifested through microbiota transplantation. Similar results have been observed in human fecal microbiota transplantation (FMT) studies, providing further evidence that the relationship between the microbiota and metabolic traits can be independent on the host’s genetic background (Hemachandra et al., 2025). In addition, the study in agricultural animals, such as pigs, dietary intervention can change the abundance of specific microbial communities, such as *Ruminococcus*, *Prevotella*, *Spirochaeta,* and *Mycobacterium*, which are closely related to fat deposition (Hu et al., 2024; Ma et al., 2024).

These studies collectively demonstrate a functional interaction between the gut microbiota and the host. Acting as key modulators, the gut microbiota integrates genomic information and environmental exposure signals to jointly regulate lipid metabolism. The regulatory effect of the gut microbiota on fat deposition is largely mediated by its rich repertoire of metabolites (Geng et al., 2022). These microbial metabolites, including SCFAs, bile acids, BCAAs, and TMAO, are important signaling molecules that connect microbial ecological shifts to host physiological changes (Agus et al., 2021; Wang C. et al., 2024). They mediate fat deposition through at least two key mechanisms: First, microbial metabolites directly target core host signaling pathways involved in lipogenesis and lipid storage, such as PPARγ and C/EBPα (Wang et al., 2022; Luo et al., 2025). Second, microbial metabolites play a crucial role in epigenetic regulation, a mechanism that has garnered significant interest in recent years (Nshanian et al., 2025; Sharma et al., 2025). For instance, metabolites, such as SCFAs can modify histone acetylation and DNA methylation, thereby exerting long-term effects on the expression of genes related to fat metabolism.

These findings suggest that targeted regulation of the gut microbiota may be a promising therapeutic strategy to ameliorate obesity-related phenotypes by restoring intestinal homeostasis and metabolic function (Lee et al., 2025; Shi et al., 2025). These mechanistic insights establish the gut microbiota and its metabolites as promising targets for intervening in fat deposition. Interventions targeting the microbiota, including prebiotics, probiotics, symbiotics, postbiotics, and fecal microbiota transplantation, have shown promising results in both clinical and animal models (Soundharrajan et al., 2020; Zheng W. et al., 2024; Yang L. et al., 2025). This review synthesizes current understanding of the roles of gut microbial metabolites in regulating fat deposition, with a focus on their downstream signaling pathways and epigenetic mechanisms. Furthermore, we summarize the evidence from multi-species research to evaluate the translational potential of various intervention strategies and to discuss the prevailing challenges and future directions in precision nutrition and micro-ecological interventions.

## 2 Association of gut microbiota with host fat deposition

### 2.1 Gut microbiota regulates host energy metabolism

The gut microbiota plays a pivotal role in regulating fat accumulation by influencing energy metabolism and lipid storage. Early evidence from GF mice models provided critical insights. Compared to GF mice, conventionally raised mice exhibited ~40% higher body fat percentage and ~47% greater fat accumulation around reproductive organs, despite having lower food intake. When GF mice were colonized with conventional microbiota, their body fat increased by 60% within just 14 days, accompanied by significant upregulation of key lipogenic genes, including carbohydrate response element binding protein (*ChREBP*) and sterol response element binding protein 1 (*SREBP-1*) (Bäckhed et al., 2004). These findings suggest that gut microbiota enhances dietary energy harvest and reprograms host metabolic pathways to promote fat storage.

The causal relationship between gut microbiota and obesity phenotypes has been validated in multiple models. Transplanting the fecal microbiota of C57BL/6 mice deficient in activation-induced cytidine deaminase into GF mice resulted in a more significant increase in the body weight of the recipient mice compared to the use of fecal microbiota from wild-type mice for transplantation (Pearson et al., 2022). However, the process by which the microbiota regulates the obesity phenotype is influenced by dietary components (Rodriguez et al., 2019). For instance, when the microbiota from the *ob/ob* mice was transplanted into recipient mice fed a low-fat and high-fiber diet, the transmission of this obesity phenotype was not strong (Ridaura et al., 2013).

### 2.2 Obesity-associated gut microbiota

The composition and diversity of the gut microbiota are strongly associated with variations in individual fat deposition (Lai et al., 2024). Early studies found that the structure of the gut microbial community in obese individuals was changed. For example, the ratio of Firmicutes to Bacteroidetes increased (Moreno-Indias et al., 2014). This change may be related to the enrichment effect of high-fat and high-sugar dietary patterns on specific bacterial groups (Mamun et al., 2025). Long-term unbalanced nutrient intake will alter the intestinal environment, thereby selectively promoting the proliferation of certain microorganisms. Subsequent research has identified a growing number of bacteria associated with obesity. Specifically, *Lactobacillus paracasei* (*L. paracasei*) (Yao et al., 2019), *Lactobacillus plantarum* (*L. plantarum*) (Lee et al., 2021), *Bifidobacterium longum* (Lee et al., 2025), and *Clostridium butyricum* (*C. butyricum*) (Chen et al., 2020) have been shown to be negatively correlated with fat deposition, whereas *Methanobrevibacter smithii* (Million et al., 2012) and *Prevotella copri* (Chen et al., 2021) are positively correlated with it. Nevertheless, the biological insight gained from merely cataloging bacterial species remains limited.

In addition to correlation studies, the causal role of specific gut microorganisms in obesity has been increasingly demonstrated. For instance, *Akkermansia muciniphila* (*A. muciniphila*), a highly promising next generation “candidate probiotic,” has been found to be negatively correlated with obesity, type 2 diabetes, and hypertension (Depommier et al., 2019). This bacterium can improve intestinal barrier function by increasing the thickness of the intestinal mucus layer, thereby alleviating metabolic endotoxemia and low-grade inflammation. This protective process is partially dependent on the interaction between its outer membrane protein Amuc_1,100 and Toll-like receptor 2 (TLR2) (Zheng et al., 2023). Moreover, *A. muciniphila* also regulates host inflammatory response and glucose homeostasis by stimulating the production of endogenous cannabinoids in the intestine (Ghaderi et al., 2022). Other studies have shown that pasteurized *A. muciniphila* exerts anti-obesity effects through multiple mechanisms. These include upregulation of tight junction proteins Zonula occludens-1 (ZO-1) and Occludin to enhance intestinal barrier integrity, SCFA enrichments to promote the growth of beneficial bacteria, increased levels of SCFAs and glucagon-like peptide-1 (GLP-1) in the blood, and the influences of key metabolic processes by regulating the AMP-activated protein kinase/peroxisome proliferator-activated receptor alpha (AMPK/PPAR-*α*) and PPARγ signaling pathways (Yang L. et al., 2025). Thus, multiple gut bacteria can participate in obesity regulation through diverse mechanisms.

The influence of gut bacteria on fat deposition is site-specific. The small intestinal microbiota plays a crucial role in the initial digestion and absorption of dietary fat and can adapt to dietary changes to modulate lipid absorption (Delbaere et al., 2023). For instance, experiments have demonstrated that *Clostridium bifermentans* in the small intestine promotes the absorption of oleic acid and upregulates the expression of diacylglycerol O-acyltransferase 2 (*Dgat2*), a gene critical for triglyceride synthesis (Martinez-Guryn et al., 2018). This suggests that the microorganisms in the small intestine may affect the efficiency of dietary fat utilization by directly regulating the host’s lipid metabolism pathways. The large intestine, in contrast, harbors a more diverse and complex microbial community. These microbes ferment carbohydrates to produce metabolites, such as SCFAs, thereby modulating systemic energy metabolism, inflammatory responses, and fat storage (Mukhopadhya and Louis, 2025). For example, *C. butyricum* can increase serum SCFA levels and enhance the carbohydrate-fermenting capacity of the gut microbiota, thereby slowing host fat accumulation (Ma et al., 2024).

Furthermore, the functional characteristics of obesity-related gut microbiota vary considerably across species and even strains, reflecting the complexity of host-microbe metabolic interactions. For instance, Crovesy et al. (2017) reported that *L. paracasei* is negatively correlated with obesity, whereas *Lactobacillus reuteri* and *Lactobacillus gasseri* (*L. gasseri*) are positively correlated. This functional heterogeneity arises from differences in gene content, metabolic potential, and host-interaction strategies among different species. Even within the same species, the functions of different strains can diverge substantially. For example, *Lactobacillus reuteri* ZJ617 (Ma et al., 2025) can improve metabolic abnormalities induced by a high-fat diet (HFD), while other *Lactobacillus reuteri* strains are positively correlated with obesity, highlighting the critical importance of strain-level specificity in functional assessments (Million et al., 2012).

The gut microbiota is a key determinant of host fat deposition and the development of obesity, mediated by its diverse composition, site-specific effects, and species−/strain-dependent functional differences. From early correlative studies of community structure to current mechanistic analyses of specific bacteria, such as *Akkermansia*, research have made significant strides in uncovering the crucial role of microbes in metabolic health. Future research should aim to integrate microbial spatial localization, functional gene modules, and host interaction networks to elucidate their complex roles in the pathogenesis of obesity, thereby paving the way for novel probiotics and microbiota-guided interventions for metabolic diseases.

### 2.3 Factors resulting in the heterogeneity of obesity-associated microbiota identified in different studies

The above heterogeneity can be attributed to several key factors. First, there are significant differences in microbial composition at different sampling points, which may affect the consistency of the identified obesity-related microbial taxa. Second, relying only on 16S rRNA gene sequencing to identify the genus-level taxa often conceals the functional heterogeneity of different species and strains in the same genus (Yang M. Q. et al., 2024). For example, *Prevotella* contains a variety of strains, which play a completely different role in metabolism and inflammation (Lu et al., 2018). Third, the genetic background of the host significantly affects the structure and function of the gut microbiota, thus shaping the immune response, metabolic spectrum and the interaction between microorganisms and the host (Zhernakova et al., 2024). Despite these complexities, there is broad consensus that gut microbial metabolites and their downstream signaling pathways are central to fat deposition. This consensus underscores the utility of multi-omics strategies for elucidating host-microbiota synergistic adaptation mechanisms and developing precise interventions for metabolic health.

## 3 Microbial metabolites: key mediators in the regulation of lipid metabolism

The gut microbiota can metabolize both exogenous nutrients and endogenous host-derived substrates, resulting in the production of various bioactive metabolites. These metabolites are not only by-products of microbial fermentation, but also crucial signaling molecules mediating host–microbiota interactions, and play central roles in regulating lipid metabolism (Jyoti and Dey, 2025). Accumulating evidence have suggested that these microbial metabolites contribute to host energy balance, suppress inflammatory responses, and modulate oxidative stress (Ejtahed et al., 2020). In recent years, dysbiosis of the gut microbiota has been increasingly linked to the pathogenesis of obesity and related metabolic disorders (Zhang et al., 2023), particularly through disrupting the biosynthesis of key metabolites, such as SCFAs (Mukhopadhya and Louis, 2025), tryptophan-derived compounds (Xue et al., 2023), and secondary bile acids (Tu et al., 2022). These small molecules act as molecular bridges connecting microbial functionality to host metabolic phenotypes by targeting specific signaling pathways and altering the intestinal microenvironment.

### 3.1 SCFAs

SCFAs are the principal metabolites generated by the fermentation of non-digestible carbohydrates by gut microbiota. SCFAs mainly include acetate, propionate, and butyrate, typically in a molar ratio of approximately 60:20:20 (May and den Hartigh, 2023). SCFAs regulate intracellular energy metabolism through several signaling cascades. Both acetate and butyrate activate the AMPK pathway, inhibit acetyl-CoA carboxylase (ACC), and promote *β*-oxidation of fatty acids (Lee et al., 2021; Yang L. et al., 2025). Butyrate also functions as an HDAC inhibitor that enhances *PPARγ* expression and promotes a shift of adipocytes toward a more oxidative phenotype (Yang J. et al., 2025). Although propionate and butyrate are present at lower concentrations in the peripheral circulation compared to acetate, their combined effects on adipose tissue, liver, and skeletal muscle significantly influence glucose and lipid metabolism, thereby maintaining systemic metabolic homeostasis (Luo et al., 2022).

In human studies, SCFAs, such as propionate and acetate have been shown to stimulate the secretion of gut hormones like GLP-1 and peptide YY (PYY), reducing energy intake and attenuating weight gain (Bastings et al., 2023). In animal production, SCFAs have demonstrated similar metabolic benefits. In pigs, exogenous SCFAs administration downregulates the expression of adipogenic genes and activates lipolytic pathways, which leads to reduced backfat thickness and improved meat quality, highlighting their potential in precision nutritional strategies for livestock (Jiao et al., 2020).

The effects of SCFAs are pleiotropic and often context-dependent, arising from a complex interplay of factors, such as dose, cell type, receptor expression, and metabolic environment. For instance, SCFAs promoted adipocyte differentiation and lipid accumulation in 3 T3-L1 preadipocytes *in vitro* (Yu et al., 2018), while their supplementation prevented or alleviated high-fat diet (HFD)-induced obesity in multiple *in vivo* animal models (Shimizu et al., 2019; Jiao et al., 2021). To reconcile this apparent contradiction, several factors must be considered. First, the concentration of SCFAs varies significantly across physiological compartments, leading to divergent effects. At the high millimolar (mM) concentrations typically in in vitro studies, SCFAs function as histone deacetylase inhibitors and directly upregulate lipogenic genes like fatty acid binding protein 4 (*FABP4*) and fatty acid synthase (*FAS*) via epigenetic mechanisms, thereby promoting adipogenesis (Yu et al., 2018). In contrast, at the lower concentrations found in systemic circulation, SCFAs primarily signal through G protein-coupled receptors (GPCRs). For example, they activate GPCRs, such as G protein-coupled receptor 41 (GPR41) and G protein-coupled receptor 43 (GPR43) on intestinal endocrine cells and stimulate the secretion of satiety hormones, e.g., GLP-1 and PYY, which suppress appetite and reduce fat intake (Jiao et al., 2020; Jiao et al., 2021). Second, SCFAs must act through receptors, primarily GPCRs, whose expression profiles vary by tissue and cell type. In adipose tissue, SCFAs may directly regulate lipogenesis via HDAC inhibition. In intestinal L cells and hepatocytes, they signal through receptors, including GPR41, GPR43, and olfactory receptor 78 (Olfr78), and activate downstream pathways like AMPK to inhibit gluconeogenesis and promote fatty acid oxidation (Yoshida et al., 2019; Li et al., 2021). In the vagus nerve, SCFAs activate afferent neurons to regulate appetite and energy balance (Goswami et al., 2018). Thus, the net effect of SCFAs is ultimately determined by the specific target tissue and its unique receptor expression profile. Additionally, the host’s overall metabolic state is a critical variable. In HFD-induced obese mice, SCFA supplementation demonstrates a potential ameliorative effect by correcting gut microbiota dysbiosis, increasing energy expenditure, and improving insulin sensitivity. These systemic benefits override their direct potential to promote adipogenesis (Shimizu et al., 2019; Mandaliya et al., 2021). In contrast, in healthy contexts, the effects of exogenous SCFAs may be subtle, potentially including slightly increased energy absorption due to colonic epithelial utilization of butyrate (Hamer et al., 2008).

In summary, the impact of SCFAs on fat deposition is not a binary of “promotion” or “inhibition,” but a dynamic equilibrium shaped by dose, gut site of action, receptor distribution, and host metabolic status. Therefore, categorizing SCFAs as “good” or “bad” simply oversimplifies their complex biological functions. Future research and therapeutic applications, such as prebiotics or SCFA supplements for obesity, must account for these variables to achieve precise modulation.

### 3.2 Bile acids

Bile acids are amphipathic steroid molecules synthesized in the liver from cholesterol. Their biosynthesis, transformation, and signaling functions are intricately modulated by the gut microbiota (Collins et al., 2023). The primary bile acids mainly including cholic acid (CA) and chenodeoxycholic acid (CDCA) are conjugated with glycine or taurine to form bile salts, which are stored in the gallbladder. Following food intake, gallbladder contraction releases bile acids into the small intestine, where they facilitate the emulsification of dietary lipids and the absorption of fat-soluble vitamins (Voronova et al., 2020). Approximately 95% of bile acids are reabsorbed in the terminal ileum and transported back to the liver via the portal vein, forming a tightly regulated enterohepatic circulation. The remaining bile acids enter the colon, where they are either excreted or modified by the gut microbiota (Chiang and Ferrell, 2019). The modification of bile acids by microorganisms cannot be achieved without bile salt hydrolase (BSH). BSH is a key enzyme produced by commensal gut bacteria throughout the intestinal tract (Dong et al., 2025). It catalyzes the deconjugation of primary bile acids, such as taurocholic acid, by cleaving off their glycine or taurine side chains, specifically through hydrolysis of the C-24 N-acyl bond, thereby generating free bile acids, such as cholic acid (CA) and chenodeoxycholic acid (CDCA) (Ridlon et al., 2016; Rimal et al., 2024). This deconjugation is a critical first step in microbial bile acid metabolism. The resulted free bile acids then serve as substrates for further microbially encoded transformations, including 7α-dehydroxylation, dehydrogenation, and dihydroxylation, and ultimately generated secondary bile acids with broad effects on host physiology and the gut microbial ecosystem itself (Wise and Cummings, 2022). By governing the size of the deconjugated bile acid pool, BSH activity directly shapes the overall bile acid composition, which in turn modulates host lipid metabolism. Intestinal overexpression of BSH, for instance, can significantly reshape the plasma bile acid profile and consequently regulate the expression of key host genes across multiple metabolic pathways. These include genes involved in fat metabolism like *PPARγ* and angiopoietin-like 4 (*Angptl4*), and genes governing cholesterol homeostasis, such as ATP-binding cassette subfamily G member 5/8 (*Abcg5/8*) (Joyce et al., 2014). Collectively, these changes can lead to reduced weight gain and improved plasma cholesterol levels (Joyce et al., 2014). As a specific example, *Lactobacillus texensis* modulates host cholesterol metabolism by altering bile acid cycling via its BSH activity (Hou et al., 2020).

Disruptions in bile acid metabolism are frequently observed in metabolic disorders. HFD increase the production of taurocholic acids (TCAs) and favor the expansion of sulfite-reducing bacteria, such as *Bilophila*, which impair intestinal barrier integrity and promote hepatic steatosis (Devkota et al., 2012). Moreover, altered bile acid composition may exacerbate metabolic dysfunction by promoting the phosphorylation of tight junction proteins and increasing gut permeability. Clinical evidence also supports a role of bile acids in metabolic regulation. Oral supplementation with CDCA, for example, enhances mitochondrial uncoupling in human brown adipocytes and increases whole-body energy expenditure. Post-bariatric surgery changes the bile acid pool, which was characterized by elevated levels of secondary bile acids and might contribute to the observed improvements in metabolic health (Tu et al., 2022). Bile acids have been also utilized as feed additives in animal production. In swine models, dietary supplementation with bile acids has been shown to promote growth performance, increase daily weight gain and feed intake, and reduce backfat thickness. For example, incorporating 0.025% porcine DCA into a diet containing 25% aged Japonica brown rice improved meat quality and decreased fat deposition in minipigs (Wang C. et al., 2024). The biological effects of bile acids are primarily mediated through their receptors. In a porcine model of non-alcoholic fatty liver disease, impaired (bile acid-activated Farnesoid X Receptor) FXR signaling disrupted downstream metabolic and inflammatory pathways (Maj et al., 2023). TGR5 activation, on the other hand, promotes mitochondrial uncoupling and thermogenesis in brown adipose tissue. This improves glucose tolerance and energy expenditure (Velazquez-Villegas et al., 2018). Additionally, porcine hyocholic acid (HCA) has been shown to enhance intestinal GLP-1 secretion through simultaneous TGR5 activation and FXR inhibition, thereby contributing to energy balance and insulin sensitivity (Zheng et al., 2021).

Overall, bile acids facilitate lipid digestion and absorption while also acting as key signaling molecules that regulate systemic metabolism. Their biosynthesis, transformation, and receptor-mediated activity are profoundly influenced by the gut microbiota. Through the FXR and TGR5 signaling pathways, bile acids modulate energy expenditure, lipid accumulation, and inflammation. Their roles in obesity, metabolic syndrome, and non-alcoholic fatty liver disease (NAFLD) have garnered increasing attention, and they exhibit considerable potential as metabolic regulators in livestock production. Future research should further elucidate the dynamic interplay among bile acids, gut microbes, and host metabolism to develop targeted interventions for metabolic diseases and precision nutrition in animal production.

### 3.3 Tryptophan and indole-derived metabolites

Tryptophan is an essential aromatic amino acid primarily obtained through dietary intake. In both host and gut microbiota, tryptophan undergoes transformation via several metabolic pathways, resulting in the production of diverse bioactive metabolites (Lu Z. et al., 2025). Three major tryptophan metabolic pathways include the kynurenine (KYN) pathway, the serotonin (5-hydroxytryptamine, 5-HT) pathway, and the indole pathway. In mammals, over 95% of Tryptophan is catabolized through the KYN pathway, generating a range of signaling molecules with immunometabolic functions. One such metabolite, kynurenic acid (KYNA), promotes thermogenesis in adipose tissue and suppresses HFD-induced obesity by activating G-protein coupled receptor 35 (GPR35) (Agudelo et al., 2018). The rate-limiting enzyme in this pathway is indoleamine 2,3-dioxygenase 1 (IDO1), which is notably upregulated in inflammatory states. Elevated IDO1 activity and increased circulating KYN levels have been strongly associated with insulin resistance and metabolic dysfunction in obesity (Rojas et al., 2021). Pharmacological or genetic inhibition of IDO1 has been shown to improve insulin sensitivity and restore metabolic balance. The second pathway involves the biosynthesis of 5-HT. Tryptophan is first converted into 5-hydroxytryptophan (5-HTP), which is subsequently decarboxylated to form 5-HT. While central 5-HT is involved in appetite regulation via modulation of hypothalamic circuits, peripheral 5-HT is predominantly synthesized in the gastrointestinal tract, accounting for over 80% of systemic 5-HT levels (Maffei, 2020). The gut microbiota plays a crucial role in modulating peripheral 5-HT production. Certain species, such as *Bacillus subtilis*, can stimulate enterochromaffin cells to synthesize 5-HT by producing SCFAs and basic amino acids (He et al., 2021). Functionally, peripheral 5-HT suppresses thermogenic activity in brown adipose tissue and promotes lipid accumulation, thereby contributing to obesity development. The third pathway involves direct microbial metabolism of tryptophan into various indole derivatives, including indole-3-propionic acid (IPA) and indole-3-acetic acid (IAA). These metabolites primarily serve as ligands for the aryl hydrocarbon receptor (AhR), which plays critical roles in maintaining intestinal barrier integrity, modulating immune responses, and regulating lipid metabolism (Zheng et al., 2025). In metabolic disorders, the gut microbiota’s capacity to produce indoles is often diminished, resulting in reduced levels of protective molecules, such as GLP-1 and Interleukin-22 (IL-22), and subsequently increased intestinal permeability and hepatic steatosis (Natividad et al., 2018). Interventions with AhR agonists or colonization with AhR ligand-producing probiotics (e.g., *Lactobacillus rohita*) have demonstrated efficacy in restoring intestinal barrier function and ameliorating metabolic dysregulation.

In summary, tryptophan metabolism is a central node in gut microbiota–host crosstalk. The KYN pathway contributes to immune modulation and thermogenesis, the 5-HT pathway regulates appetite and energy balance, and the indole pathway safeguards intestinal and metabolic homeostasis. Dysregulation of these pathways is frequently implicated in obesity and metabolic diseases. A comprehensive elucidation of the distinct roles of each tryptophan metabolic branch will help identify novel molecular targets and facilitate the development of precision interventions for metabolic disorder prevention and treatment.

### 3.4 LPS

LPS are major structural components of the outer membrane of Gram-negative bacteria and represent a classic class of microbial-associated molecular patterns (MAMPs). In 2007, Cani et al. first introduced the concept of metabolic endotoxemia, highlighting that elevated intestinal LPS levels can induce systemic and chronic low-grade inflammation which is a key trigger of obesity and metabolic disorders (Cani et al., 2007).

Under physiological conditions, the intestinal barrier comprising of tight junction proteins, antimicrobial peptides, and a protective mucus layer effectively prevents LPS translocation from the gut lumen into systemic circulation. However, this barrier integrity can be compromised by several metabolic stressors, including HFD, excessive alcohol intake, obesity (Suriano et al., 2021), hyperglycemia, and low dietary fiber intake. These factors reduce the expression of tight junction proteins, suppress antimicrobial peptide production, alter mucus structure, and collectively increase intestinal permeability. This facilitates the entry of LPS into the bloodstream and initiates inflammatory cascades (Paone and Cani, 2020). In addition to weakening the intestinal barrier, HFD can also promote LPS production at the source by increasing the abundance of Gram-negative bacteria within the gut microbiota. Upon entering the circulation, LPS first targets the liver and induces the overexpression of tumor necrosis factor-alpha (*TNF-α*) and chemokines, which in turn promote neutrophil infiltration, hepatocellular injury, and hepatic steatosis. *In vivo* studies have demonstrated that LPS administration significantly upregulates pro-inflammatory gene expression, including interleukin-6 (*IL-6*), *TNF-α*, interleukin-1 beta (*IL-1β*), and plasminogen activator inhibitor-1 (*PAI-1*) in adipose tissue, liver, and skeletal muscle (Henao-Mejia et al., 2012). In genetically obese models, such as *ob/ob* mice and fa/fa rats, low-dose LPS exposure accelerates the progression of steatohepatitis, while LPS antagonists (e.g., polymyxin B) alleviate hepatic lipid accumulation, further supporting its pathogenic role (Pappo et al., 1991).

In livestock, LPS exerts similar pro-inflammatory effects. In growing pigs, LPS exposure alters the expression of key genes involved in lipid metabolism, transport, and distribution within adipose and muscle tissues (Chen et al., 2021). Additionally, LPS suppresses fat mobilization by downregulating adipose triglyceride lipase (*ATGL*) activity during lipolysis.

LPS primarily exerts its immunometabolism effects through activating the Toll-like receptor 4 (TLR4) signaling pathway in conjunction with its co-receptor CD14. This activation inhibits the differentiation of preadipocytes into mature adipocytes by suppressing the expression of master adipogenic transcription factors, including PPARγ and C/EBPα. Moreover, LPS-induced pro-inflammatory cytokines (e.g., TNF-α) enhance the inflammatory microenvironment and inhibit adipogenesis via activation of the WNT/β-catenin/TCF4 signaling axis. LPS also disrupts adipokine secretion profiles and upregulates leptin, adiponectin, and apolipoproteins, thereby interfering with both energy homeostasis and immune function (Than et al., 2012).

Overall, LPS, as a key inflammatory mediator derived from gut microbiota, contributes to intestinal barrier dysfunction, TLR4 pathway activation, impaired adipogenesis, and systemic inflammation. It serves as a critical molecular link between gut dysbiosis and the development of metabolic diseases. Deciphering the tissue-specific signaling mechanisms of LPS in adipose tissue, liver, and skeletal muscle will benefit to identify novel therapeutic targets for metabolic syndrome and facilitate the development of precision strategies aiming at mitigating gut-derived inflammation.

### 3.5 BCAAs

BCAAs, including valine (Val), isoleucine (Ile), and leucine (Leu) are essential amino acids that play critical roles in protein synthesis, glucose and lipid metabolism, and the regulation of insulin signaling pathways. They are key contributors to the maintenance of host metabolic homeostasis. Recent studies have revealed that the bioavailability and systemic effects of BCAAs are not solely determined by dietary intake and host metabolism, but are also significantly influenced by the metabolic activity of the gut microbiota (Yoneshiro et al., 2019). In animal models, moderate supplementation with BCAAs has been shown to enhance the proliferation of beneficial bacterial genera such as *Akkermansia* and *Bifidobacterium*, while suppressing the growth of potentially pathogenic microbes like Enterobacteriaceae, thereby improving gut microbial composition (Yang et al., 2016).

Existing studies have revealed the complex dual roles of BCAAs in metabolism. Moderate supplementation may be beneficial, whereas excessive intake poses health risks. However, a consensus on defining the precise dosage range remains elusive (de la Oa et al., 2020). Some observational studies and short-term interventions have offered preliminary insights into potential benefits of “moderate” intake. For instance, one study reported that individuals consuming approximately 15 grams of dietary BCAAs per day had a lower risk of overweight or obesity compared to those consuming around 12 grams (Qin et al., 2011; Li et al., 2015). Additionally, among exercising populations, an eight-week supplementation of 14 grams of BCAAs daily demonstrated positive effects on fat loss and muscle gain (Stoppani et al., 2009). However, it is important to note that these studies are often limited by small sample sizes, suboptimal control group designs, and insufficient documentation of subjects’ background diets (Spillane et al., 2013; Luan et al., 2025). Consequently, such dosage data, e.g., 12–15 grams per day, should be regarded as preliminary references, falling short of the evidence strength required for universal dietary recommendations (Martinho et al., 2022).

In contrast to the ambiguity in human studies, animal experiments provide more precise and mechanistic evidence regarding the consequences of “excessive” BCAA intake. In a weaned piglet model, BCAA intake at 150% of the National Research Council (NRC) recommendations reduced serum adiponectin levels, disrupted lipid metabolism, and induced abnormal m6A RNA methylation modifications associated with insulin resistance (Heng et al., 2020). More in-depth research has shown that increasing the dietary standardized ileal digestible leucine-to-lysine ratio to 186% significantly reduces feed intake, while a ratio of 353% causes severe amino acid imbalance, particularly a decrease in brain serotonin levels, further suppressing appetite and growth performance via overactivation of the mechanistic target of rapamycin (mTOR) signaling pathway (Cemin et al., 2019). These findings clearly demonstrate that, in controlled models, “excess” corresponds to a quantifiable threshold. However, direct extrapolation of precise doses from animal studies to humans remains highly challenging. Interindividual differences in basal metabolic rate, physical activity levels, and gut microbiota composition complicate the establishment of a unified safety threshold for human populations.

In summary, a clearly defined cross-species applicable dosage range for “moderate BCAAs” has not been established in either humans or pigs. Future research should further elucidate the mechanisms through which BCAAs influence metabolic health via gut microbiota regulation, amino acid competitive transport, and m6A epigenetic modification, to enable more precise application of BCAAs in modulating fat deposition.

### 3.6 TMAO

TMAO is a gut microbiota–dependent hepatic metabolite that plays an emerging role in lipid metabolism and cardiometabolic health. It is produced through a well-defined two-step process: dietary nutrients such as choline, phosphatidylcholine, and L-carnitine are first converted by gut microbiota into trimethylamine (TMA), which is then absorbed into the portal circulation and oxidized into TMAO in the liver by the enzyme flavin-containing monooxygenase 3 (FMO3).

Multiple studies have reported significantly elevated plasma TMAO levels in individuals with obesity, type 2 diabetes, and cardiovascular diseases (Shan et al., 2017). Its role in regulating lipid metabolism has garnered substantial interest. For example, Gao et al. (2014) found that TMAO increased hepatic total cholesterol and triglyceride levels in mice, while concurrently decreasing their plasma concentrations, suggesting that TMAO may influence lipid partitioning between tissues. Similarly, Koeth et al. (2013) demonstrated that dietary L-carnitine supplementation exacerbated atherosclerosis in mice, whereas direct TMAO administration lowered plasma cholesterol levels, indicating its context-dependent regulatory effects. Moreover, in animal production, TMAO alters ileal microbial community structure and influences acetate production, which subsequently modulates fat distribution and fatty acid composition in fattening pigs (Zha et al., 2024). These findings have revealed the correlation between TMAO and lipid metabolism in both humans and animals.

The association between circulating TMAO levels and obesity is critically dependent on the expression and activity of host hepatic and adipose flavin monooxygenase 3 (*FMO3*) (Shanmugham et al., 2023). As the rate-limiting enzyme converting trimethylamine (TMA) to TMAO, *FMO3* expression is a primary determinant of systemic TMAO homeostasis (Schugar et al., 2017; Ganapathy et al., 2025). For instance, Schugar et al. demonstrated that knocking down *FMO3* with antisense oligonucleotides prevented TMAO formation and ameliorated obesity and white adipose tissue dysfunction, even under a high-choline diet. This provides direct evidence for a causal role of *FMO3* in obesity pathogenesis, rather than TMAO being a mere passive correlate (Schugar et al., 2017). Further research by Ganapathy et al. revealed that *FMO3* upregulation in adipocytes is a significant source of TMAO under aging and metabolic stress. This adipocyte-derived TMAO promotes adipose tissue inflammation and dysfunction via autocrine/paracrine signaling, whereas adipocyte-specific *FMO3* knockout reduces both local and circulating TMAO and improves metabolic phenotypes (Ganapathy et al., 2025). Collectively, these mechanistic studies establish *FMO3* activity as the core hub integrating dietary precursors, gut microbial metabolism, and the ultimate pathological effects of TMAO.

The causal role of *FMO3* is supported by interventional studies, whether TMAO itself is an independent pathogenic driver or a biomarker in human obesity requires further clarification (Caradonna et al., 2025). TMAO levels are influenced not only by dietary precursors but also by host *FMO3* activity and gut microbial composition. Therefore, future research should delineate the specific contributions of *FMO3* to TMAO generation and its precise relationship with obesity to fully elucidate the causal mechanisms in this pathway. In summary, TMAO is a key metabolite of the gut microbiota-liver axis, whose production depends on microbial precursors and host *FMO3* activity. The tight interdependence between gut microbiota and host *FMO3* underscores TMAO’s role as a central mediator of host-microbe metabolic crosstalk. Further research is warranted to clarify its concentration-dependent tissue effects, inter-individual variability, and potential as a target for nutritional intervention in both human metabolic diseases and animal production systems. Table 1 summarizes the sources, functions, and metabolic impacts of key microbiota-derived metabolites in obesity.

**Table 1:** Metabolites in obesity: sources, functions, and their impact on obesity.

Metabolite	Main sources	Main functions	Changes in obesity
SCFAs (acetate, propionate, butyrate)	Colonic fermentation of dietary fibers by *Ruminococcaceae*, *Clostridium*	Inhibit lipogenesis, promote β-oxidation, enhance gut barrier, anti-inflammation	↓ Levels; ↓ SCFA-producing taxa; ↑ colonic pH (Ke et al., 2019)
Bile acids (CA, CDCA, DCA, LCA)	Primary: Hepatic synthesis. Secondary: Microbial BSH activity (*Lactobacillus*, *Bacteroides*).	Lipid digestion, cholesterol metabolism, thermogenesis, GLP-1 secretion	↑ Taurocholic acid; ↓ FXR signaling (Collins et al., 2023)
Tryptophan derivatives (KYNA, IPA, 5-HT*)	Microbial metabolism (*Lactobacillus*, *Bacillus*); 5-HT: Host enterochromaffin cells	Barrier integrity, lipid metabolism, serotonin signaling	↓ Indole derivatives; ↑ 5-HT metabolites (linked to insulin resistance) (Xue et al., 2023)
LPS	Gram-negative bacteria (Enterobacteriaceae)	Pro-inflammatory, adipocyte dysfunction, barrier disruption	↑ Plasma LPS (metabolic endotoxemia); ↑ gut permeability (Cani et al., 2008)
BCAAs (valine, isoleucine, leucine)	Dietary protein; microbial metabolism (*Prevotella*, *Butyrivibrio*)	Protein synthesis, insulin regulation, thermogenesis	↑ Plasma BCAAs; ↓ microbial BCAAs catabolism (Arany and Neinast, 2018)
TMAO	Microbial conversion of choline/carnitine (Firmicutes); hepatic FMO3 oxidation	Cholesterol transport, foam cell formation	↑ Levels correlate with obesity risk (Dehghan et al., 2020)

### 3.7 Role of microbial metabolites in host epigenetic regulation

Gut microbiota continuously communicates with the host and transmits signals through a diverse metabolite. These metabolites not only serve as energy sources or signaling molecules, but can also directly enter the nucleus to precisely modulate gene expression. The underlying mechanisms, including DNA methylation, histone modification, non-coding RNA regulation, and chromatin remodeling, do not alter the DNA sequence itself but can exert long-term control over which genes are activated or silenced. This establishes a dynamic bridge connecting diet, gut microbiota, and host genes (Wu et al., 2023). Recent research indicates that microbial metabolites influence host epigenetics through at least three primary avenues: first, by regulating the activity of epigenetic enzymes; second, by providing essential substrates such as methyl or acetyl groups; and third, by modifying chromatin accessibility. Consequently, the host’s gene expression profile is reshaped, and some of these epigenetic modifications can be inherited by subsequent generations.

These insights have advanced our understanding of how the gut microbiota influences fat accumulation and obesity and have spurred the emergence of a new research paradigm: the “microbiota-metabolite-epigenome axis.” This conceptual framework is rapidly gaining traction as a promising target for both research and therapeutic intervention in metabolic diseases (Lin et al., 2024; Sharma et al., 2025). While most current research has focused on short-chain fatty acids (SCFAs) and tryptophan metabolites, accumulating evidence indicate that a broader spectrum of gut microbial metabolites can influence host epigenetic regulation. As integral components of the metabolic network, these metabolites play crucial roles in maintaining systemic metabolic homeostasis. Although the evidence is not yet exhaustive, it is increasingly clear that microbial metabolites function as bona fide epigenetic regulators, introducing an additional layer of regulation across diverse metabolic pathways.

#### 3.7.1 SCFAs

SCFAs modulate the host’s epigenetic landscape through multiple mechanisms, including the regulation of histone modifications, DNA and RNA methylation, chromatin remodeling, and non-coding RNA expression (Nshanian et al., 2025). In essence, they function not only as energy substrates but, more accurately, as signaling molecules that directly fine-tune transcriptional programs.

By inhibiting histone deacetylase (HDAC) activity, SCFAs can suppress the proliferation of tuft cells, the generation of Th17 cells, and the activation of mast cells. These effects, in turn, modulate type 2 immune responses, inflammation, and autoimmune and allergic processes in the gut (Eshleman et al., 2024). Furthermore, SCFAs enhance mTOR pathway activity in CD8 + T cells to promote the release of anti-tumor factors (Luu et al., 2021) and can activate the histone acetyltransferase p300 (Thomas and Denu, 2021). *In vitro* experiments show that SCFAs, such as butyrate and acetate can induce various histone modifications, including crotonylation, acetylation, and propionylation (Yuan et al., 2023). Notably, SCFA levels are closely linked to alterations in DNA methylation patterns, which are associated with susceptibility to and the pathogenesis of diabetes (Guo et al., 2022). For instance, acetate and butyrate can reshape the DNA methylome of regulatory T cells and macrophages, potentially conferring protection against food allergies and cardiovascular diseases (Kaye et al., 2020). Butyrate can also downregulate the expression of the methyltransferase 3, N6-adenosine-methyltransferase complex catalytic subunit (*Mettl3*), thereby reducing the level of RNA m6A modification (Liu K. et al., 2023). Although these studies demonstrate that SCFAs can reshape the host’s epigenetic landscape, affecting both DNA and RNA methylation, the majority of evidence is derived from animal models or *in vitro* systems. Further functional studies are required to verify whether SCFAs regulate DNA or RNA methylation in an analogous manner in humans.

Beyond direct histone modifications, butyrate can regulate immune cell function and development by modulating chromatin remodeling (Yang et al., 2020). Notably, SCFAs also influence epigenetic regulation by controlling the availability of essential substrates. For instance, pentanoate increases the intracellular concentration of acetyl-CoA, a key substrate for histone acetyltransferases (Luu et al., 2019). Once integrated into the tricarboxylic acid (TCA) cycle, acetyl-CoA further influences cellular metabolic flux. While research on SCFAs and non-coding RNA (ncRNA) regulation is still emerging, current evidence underscores their significant role in this area (Majumdar et al., 2024). In summary, SCFAs exert multiple epigenetic functions by regulating HDAC activity, influencing substrate supply, and altering chromatin structure, thereby profoundly impacting metabolic regulation. The distinct roles of specific SCFAs are increasingly clear: Butyrate-induced histone acetylation promotes immune cell infiltration into adipose tissue while improving glucose metabolism and islet function (Pedersen et al., 2024). Acetate serving as a direct precursor provides acetyl groups to support histone acetylation and maintain metabolic health. Propionate, on the other hand, contributes to metabolic regulation by modulating DNA methylation of genes involved in gluconeogenesis and insulin sensitivity (Remely et al., 2014).

#### 3.7.2 Bile acids

The alterations in the gut microbiota composition can reshape the host’s bile acid profile, thereby reprogramming the host epigenome (Fan et al., 2023). For instance, tauroursodeoxycholic acid regulates histone methylation near genes controlling lipid droplet size, thereby ameliorating hepatic steatosis (Urmi et al., 2019). Additionally, gut microbiota-derived taurocholic acid enhances the binding of H3K4me1 to genes involved in glycolysis and immunosuppression, which promotes glycolytic activity in myeloid-derived suppressor cells (MDSCs) and facilitates their dissemination to the lungs (Liu et al., 2024). Furthermore, gut microbiota and bile acids act in concert to inhibit ten-eleven translocation 1 (*TET1*) activity, thereby reducing DNA hydromethylation levels in intestinal innate lymphoid cell precursors (Zhang et al., 2024). Collectively, these findings establish that bile acids function not only as digestive intermediaries, but also as key microbiota-derived signaling molecules. They profoundly influence host immune regulation and metabolic homeostasis by modulating epigenetic mechanisms, including DNA hydromethylation and histone modifications.

#### 3.7.3 Tryptophan and indole-derived metabolites

Tryptophan serves as a critical bridge connecting the host and the gut microbiota, and its microbial metabolites have emerged as key regulators of physiological function and disease (Zhu et al., 2023; Pedersen et al., 2024; Zhang et al., 2024). Although research on their epigenetic roles remains limited, this field is expanding rapidly. Emerging evidence indicate that tryptophan-derived metabolites, including indole-3-propionic acid (IPA) and indole-3-lactic acid (ILA), can modulate histone acetylation, methylation, and chromatin accessibility, thereby regulating gene expression. A prominent example is IPA which upregulates lysine demethylase 6B (*KDM6B*) to reduce H3K27me3 levels at the promoter of the mitochondrial transcription factor A (*TFAM*) gene. This promotes mitochondrial biogenesis, reduces adipose tissue inflammation, and ameliorates obesity-associated osteoporosis (Behera et al., 2021). In a separate pathway, dietary radish alleviates hyperuricemia by reshaping the gut microbiota and host metabolism, activating the nuclear factor erythroid 2-related factor 2 (Nrf2) pathway, and mediating epigenetic modifications (Wang et al., 2023). Notably, this relationship is bidirectional. Host epigenetic machinery, in turn, shapes the production of microbial metabolites. For example, the host enzyme ten-eleven translocation 2 (*TET2*) can regulate levels of indole-3-aldehyde (I3A) produced by *Lactobacillus* (Pandey et al., 2022). This establishes a feedback loop wherein the host’s epigenetic state influences the landscape of microbial metabolites, which subsequently feedback to fine-tune the host’s epigenome.

#### 3.7.4 BCAAs

Branched-chain amino acids (BCAAs) are integral to the metabolic activities of gut microbiota, it influences not only microbial energy and nutrition, but also systemic BCAA homeostasis in the host. Comparative studies between germ-free (GF) and specific pathogen-free (SPF) mice have revealed that colonization by gut microbiota lowers intestinal and systemic BCAA levels, while elevating their degradation products sand underscoring the profound impact of microbes on host amino acid dynamics (Meier et al., 2023). Beyond their nutritional role, BCAAs and their derivatives modulate epigenetic regulation. They can influence the activity of histone deacetylases (HDACs) and DNA methyltransferases (DNMTs) (Streck et al., 2021). Notably, metabolites such as isoleucine and pentanoate serve as precursors for nuclear propionyl-CoA, thereby regulating histone propionylation, a newly discovered modification whose functional roles are still being elucidated (Yang et al., 2023). Current evidence suggests that histone propionylation may regulate genes involved in energy metabolism and cell proliferation by altering chromatin architecture or recruiting specific reader proteins (Yang et al., 2023). Thus, gut microbial regulation of BCAA metabolism not only affects amino acid balance but may also orchestrate host epigenetic programs by altering the availability of key substrates for histone modifications.

#### 3.7.5 The role of microbial metabolites in microRNA-mediated regulation

Beyond the well-characterized mechanisms of histone modification and DNA methylation, microbial metabolites also participate in regulation via microRNA pathways. For example, SCFAs and polyphenolic compounds can regulate miRNA expression, thereby contributing to the prevention of obesity and improved glucose tolerance (Du et al., 2021). This suggests the existence of a “post-transcriptional” mode of communication between the host and microbiota via miRNA. Notably, fecal miRNA profiles in individuals with distinct dietary habits often correlate with specific gut microbiota composition patterns. Among these, certain miRNAs involved in lipid metabolism show strong associations with particular bacterial communities and nutrient intake (Tarallo et al., 2022). These findings position the “diet-microbiota-miRNA” signature as a potential early biomarker for metabolic diseases and offer novel insights into disease pathogenesis.

In summary, these epigenomic alterations not only regulate the expression of metabolism-associated genes but are also implicated in the development of metabolic disorders. However, our current understanding of the precise mechanisms of action of individual microbial metabolites across different metabolic states remains limited. Future research will require the application of cutting-edge technologies, such as CRISPR-based epigenetic editing, isotope tracing, and single-cell multi-omics, to elucidate these molecular mechanisms and establish causal relationships.

## 4 Core signaling pathways of host and gut microbiota interactions in fat deposition

The gut microbiota plays a central role in regulating host metabolic networks particularly through its ability to modulate fat deposition and energy homeostasis via microbiota-derived metabolites. Numerous studies have demonstrated that gut microbes not only interact with the host immune and metabolic systems through these metabolites but also influence key signaling pathways implicated in adipose tissue development and systemic metabolism. These include the Wnt/*β*-catenin, TGF-β/SMAD3, PPARγ, C/EBPα, and NF-κB pathways. These signaling axes govern adipocyte fate decisions, lipid synthesis and breakdown, metabolic homeostasis, and the pathogenesis of metabolic disorders. Given these multifaceted roles, the gut microbiota is increasingly recognized as a critical intermediary connecting environmental cues with host metabolic responses and represents a promising target for therapeutic strategies aimed at lipid regulation and metabolic disease intervention.

### 4.1 Wnt/*β*-catenin signaling pathway

The Wnt/β-catenin pathway plays a vital role in adipocyte differentiation, intestinal barrier function, and energy regulation. Wnt proteins are secreted glycoproteins that bind to receptors such as Frizzled and low-density lipoprotein receptor-related protein 5/6 (LRP5/6), triggering a signaling cascade that stabilizes cytoplasmic β-catenin. Accumulated β-catenin is translocated into the nucleus, where it interacts with *β*-catenin-T-cell factor/lymphoid enhancer-binding factor (TCF/LEF) to modulate the transcription of target genes (Huang et al., 2024).

The gut microbiota regulates the Wnt/β-catenin signaling pathway through metabolites, including bile acids and SCFAs. Specifically, FXR forms an inhibitory complex with β-catenin, attenuating its binding to TCF4 and thereby suppressing Wnt/β-catenin transcriptional activity (Zhang et al., 2019). In a distinct, potentially context-dependent mechanism, FXR agonists, such as obeticholic acid (OCA) or GW4064 have been shown to promote β-catenin nuclear translocation and the expression of downstream targeted genes, e.g., lymphoid enhancer-binding factor 1 (*Lef1*), cyclin D1 (*Ccnd1*), claudin 5 (*Cldn5*), *ZO-1* in endothelial or epithelial cells. This FXR-mediated activation enhances intestinal barrier integrity and promotes closure of the gut-vascular barrier, counteracting microbiota-driven barrier disruption, reducing bacterial translocation, and alleviating hepatic steatosis and non-alcoholic steatohepatitis (NASH) phenotypes (Mouries et al., 2019).

Butyrate, a microbial metabolite primarily produced by *C. butyricum*, has been reported to suppress Wnt pathway activity, reshape gut microbial composition, and promote the proliferation of beneficial taxa such as *Akkermansia* (Chen et al., 2020). Moreover, butyrate maintains intestinal mucosal stability through the “macrophage–Wnt–ERK1/2–MUC2” signaling axis (Liang et al., 2022). Another study demonstrated that *Lactobacillus rhamnosus* (*L. rhamnosus*) induced Wnt family member 10B (Wnt10b) secretion by CD8^+^ T cells via butyrate production, thereby inhibiting adipogenesis and simultaneously promoting bone metabolism (Tyagi et al., 2018). These findings underscore the importance of microbial-derived SCFAs in regulating the Wnt pathway across multiple tissues. *In vitro* studies further elucidate the tumor-suppressive mechanisms of butyrate. In colorectal cancer cell lines (HCT116, SW620), butyrate induces *β*-catenin degradation via activation of a lysosome-dependent autophagy pathway, independent of the adenomatous polyposis coli (APC) or *β*-catenin mutation status. This process blocks β-catenin nuclear translocation and downregulates proliferation markers. Supporting this, TCGA data reveal a negative correlation between catenin beta 1 (*CTNNB1*) and the autophagy gene autophagy related 4D (*ATG4D*), and silencing ATG4D reverses the *β*-catenin reduction and restores proliferation (Garavaglia et al., 2022). Conversely, in Huh7 cells of liver cancer, butyrate upregulates microRNA-22 (*miR-22*) expression, which inhibits SIRT1 and triggers reactive oxygen species (ROS) bursts and mitochondrial apoptosis, which were evidenced by cytochrome c release and caspase-3 activation. This is accompanied by increased phosphatase and tensin homolog (*PTEN*) and glycogen synthase kinase 3 (*GSK3*) expression, and decreased p-Akt and β-catenin levels. Critically, antagonizing miR-22 completely reverses these effects (Kumar et al., 2024). These studies collectively demonstrate that the molecular mechanisms by which butyrate inhibits the Wnt/β-catenin pathway vary highly depending on the cellular and pathological context. Together, they establish a solid mechanistic foundation for precision interventions targeting the gut microbiota and their metabolites.

### 4.2 TGF-β/SMAD3 signaling pathway

The transforming growth factor-beta (TGF-β) signaling pathway is a critical regulator of adipocyte differentiation, fibrogenesis, and metabolic disease progression (Li et al., 2017). Upon TGF-β stimulation, the intracellular SMAD3 protein becomes phosphorylated and translocates to the nucleus to regulate gene expression related to mesenchymal stem cell fate and lipid deposition. Overactivation of this pathway contributes to adipose tissue hypertrophy and metabolic dysfunction, whereas SMAD3 inhibition has been shown to reduce adipocyte size and improve systemic metabolic profiles (Tsukamoto et al., 2023).

A critical emerging paradigm in metabolic inflammation is the crosstalk between TGF-β/SMAD3 and NF-κB signaling pathways. For instance, LPS activation of TLR4 triggers the MyD88/NF-κB cascade, leading to the production of pro-inflammatory cytokines like tumor necrosis factor alpha (TNF-*α*) and IL-6 (Wang et al., 2019; Fitzgerald and Kagan, 2020). Notably, SMAD3 not only functions within the canonical transforming growth factor beta 1 (TGFβ1) pathway but also directly engages with and modulates NF-κB activity (Hogan et al., 2013). In some contexts, SMAD3 activation can potentiate NF-κB signaling. Specifically, TGFβ1 can activate inhibitor of nuclear factor kappa B kinase (IKK) to promote NF-κB signaling, and through Smad3, it enhances the expression of NF-κB-dependent genes like *TNF-α* (Hogan et al., 2013). This synergy may exacerbate inflammation-driven lipid deposition and metabolic disorders (Miao et al., 2024; Yang Y. et al., 2024). This crosstalk illustrates a mechanism whereby SMAD3 exacerbates metabolic dysfunction by amplifying NF-κB-mediated inflammation.

Gut microbiota may influence abnormal lipid metabolism through modulation of the TGF-β/SMAD3 signaling pathway (Rajani and Jia, 2018). In a mouse model of pulmonary fibrosis, reductions in *Lactobacillus* (e.g., *Lactobacillus johnsonii*, *L. gasseri*) were accompanied by increases in Verrucomicrobiales and Enterobacteriales, correlating with heightened TGF-β/SMAD3 activity (Quan et al., 2022). Conversely, hydroxy-α-sandalwood alcohol (HYA), a metabolite derived from *Lactobacillus*, inhibits SMAD3 phosphorylation and downregulates pro-fibrotic gene expression (Kasahara et al., 2023). The gut microbiota and its metabolites equally impact the NF-κB pathway. Specific probiotics, such as *L. rhamnosus*, *Clostridium butyracillus*, *Enterococcus faecalis*, and their metabolites, such as butyric acid and propionic acid, can inhibit the TLR4/MyD88/NF-κB axis and reduce pro-inflammatory cytokine production (Wang et al., 2019; Li P. et al., 2025; Srivastava and Mohanty, 2025). The documented crosstalk between TGF-β/SMAD3 and NF-κB pathways positions the gut microbiota as a master regulator at this critical intersection. Thus, probiotic interventions or microbial metabolites hold therapeutic potential by simultaneously curbing NF-κB-driven inflammation and fine-tuning the TGF-β/SMAD3 pathway, thereby ameliorating metabolic and inflammatory dysregulation (Luo et al., 2025).

### 4.3 PPARγ signaling pathway

PPARγ is a master transcription factor in adipogenesis, regulating lipid synthesis, storage, and insulin sensitivity (Sun et al., 2021). Working in concert with other PPAR subtypes (e.g., PPARα/β), it plays a vital role in maintaining metabolic homeostasis. Activation of PPARγ promotes fat accumulation, whereas its inhibition has been shown to alleviate metabolic disorders (Wu et al., 2022). Various gut microbiota–targeted interventions influence lipid metabolism by modulating the PPARγ pathway. For example, Garcinia cambogia extract improves gut microbial composition and downregulates hepatic expression of PPARγ and C/EBPα, while activating phosphorylated protein kinase A (p-PKA) and phosphorylated hormone-sensitive lipase (p-HSL), thereby promoting lipolysis (Tung et al., 2021). Probiotic strains such as *L. paracasei* BEPC22 and *L. plantarum* BELP53 reduce PPARγ expression and enhance PPARα activation, which promotes fatty acid oxidation and correlates with increased abundance of *Akkermansia* (Lee et al., 2024). Moreover, *Akkermansia muciniphila* outer membrane protein 1,100 (Amuc_1,100), a membrane protein derived from *A. muciniphila*, activates the adenylate cyclase 3/protein kinase A/hormone-sensitive lipase (AC3/PKA/HSL) pathway to stimulate lipolysis and induces a “white-to-brown” transition in adipocytes (Zheng et al., 2023).

Dietary interventions also modulate PPARγ signaling through microbiota-derived metabolites. For instance, astragalus polysaccharides inhibit hepatic lipid synthesis and promote lipolysis by shifting microbial composition, increasing SCFAs production, and suppressing the expression of SREBP-1c, FAS, C/EBPα, and PPARγ (Li et al., 2022). Similarly, folic acid supplementation enhances levels of SCFA-producing bacteria (e.g., *Alistipes*, *Oscillospira*), elevates acetic and propionic acid concentrations, and downregulates adipogenic transcription factors, thereby inhibiting adipocyte proliferation and differentiation (Liu Y. et al., 2023). Additionally, secondary bile acids inhibit the expression of PPARγ and SREBP-1c via activation of FXR and TGR5, contributing to reduced adipogenesis (Zhang et al., 2020).

### 4.4 C/EBPα signaling pathway

Members of the CCAAT/enhancer-binding protein (C/EBP) family orchestrate distinct stages of adipocyte differentiation. C/EBPβ and C/EBPδ act in early stages to induce the expression of C/EBPα and PPARγ, which drive terminal adipocyte maturation, whereas C/EBPγ and C/EBP homologous protein (CHOP function as negative regulators) (Wang et al., 2022). Notably, in the absence of PPARγ, C/EBPβ is unable to induce C/EBPα expression (Wang et al., 2022).

Gut microbiota can influence adipogenesis via the C/EBPα signaling axis. For instance, *L. plantarum* A29 downregulates PPARγ, C/EBPα, and C/EBPβ, along with their downstream targets in 3 T3-L1 cells, thereby suppressing adipocyte differentiation and lipid accumulation (Soundharrajan et al., 2020). Similarly, *Lactobacillus fermentum* CQPC06 improves microbial composition in NAFLD mice, enhances lipid oxidation (via PPARα, cholesterol 7 alpha-hydroxylase (CYP7A1), carnitine palmitoyltransferase 1 (CPT1), and lipoprotein lipase (LPL)), and downregulates adipogenic pathways (PPARγ, C/EBPα) (Mu et al., 2020). Some plant-derived compounds also modulate microbial communities and suppress the expression of C/EBPα, PPARγ, and SREBP-1, while activating AMPKα and repressing adipogenesis-related genes (Tung et al., 2020). Dietary factors also impact lipid metabolism by modulating gut microbiota. For example, glycine enhances the abundance of beneficial bacteria, inhibits the activating transcription factor 6 alpha–C/EBP homologous protein (ATF6α–CHOP) stress response pathway, and improves both inflammation and lipid metabolism (Zhang et al., 2021).

Interestingly, microbial metabolites exert bidirectional effects on this pathway. While butyrate has been shown to promote C/EBPα and C/EBP*β* expression and enhance adipocyte maturation and glucose metabolism *in vitro*, inflammatory factors like LPS can activate the C/EBPβ/asparagine endopeptidase (C/EBPβ/AEP) pathway, thereby affecting adipogenic gene expression (Fang et al., 2024). Despite its significance, the C/EBPα signaling pathway remains relatively underexplored in microbiota research, and further studies are warranted to elucidate its interaction with gut-derived metabolites and microbial taxa.

### 4.5 NF-κB signaling pathway

NF-κB is a key transcription factor involved in the regulation of inflammation, immunity, apoptosis, and metabolism (Zhao et al., 2024). In adipose tissue, NF-κB modulates fat storage and insulin sensitivity by influencing the expression of inflammation- and lipid-related genes (Lu Y. et al., 2025). The gut microbiota and its metabolites can activate NF-κB signaling primarily through Toll-like receptors (TLRs), particularly TLR4 (Fitzgerald and Kagan, 2020). Upon activation by ligands, such as LPS, TLR4 recruits the adaptor protein MyD88 to form a signaling complex known as the myddosome. This complex subsequently activates the IκB kinase (IKK) complex, which phosphorylates the inhibitory protein IκBα at serine 32 and 36, leading to its polyubiquitination and proteasomal degradation (Kuzmich et al., 2017).

**Figure 1:**
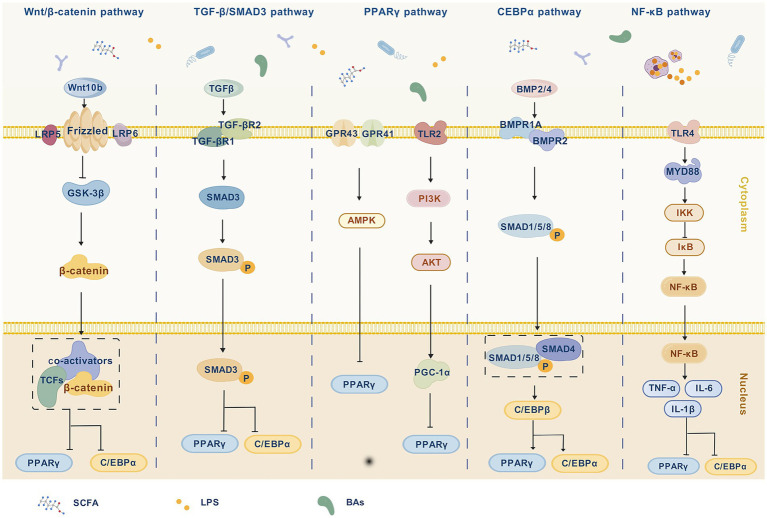
The mechanism of gut microbiota regulating fat deposition. The Wnt/β-catenin pathway activates the Frizzled and LRP5/6 receptor complex through Wnt10b, which in turn inhibits GSK-3β (glycogen synthase kinase-3β), maintaining the stability of β-catenin. Stable β-catenin is translocated from the cytoplasm to the nucleus, where it binds to TCFs and, through co-activators, co-regulates the function of β-catenin, indirectly inhibiting PPARγ and C/EBPα, thereby affecting the regulation of fat metabolism. In the TGF-β/SMAD3 pathway, TGF-β binds to its receptors TGF-βR1 and TGF-βR2, activating SMAD3. Activated SMAD3 undergoes phosphorylation (forming SMAD3-P), which then translocates to the nucleus and inhibits the expression of PPARγ and C/EBPα, thus reducing fat synthesis and deposition. GPR43 and GPR41 sense SCFAs and activate the AMPK signaling pathway, which in turn inhibits the activity of PPARγ. Additionally, TLR2, PI3K, and AKT activate PGC-1α, further inhibiting the activity of PPARγ. In the C/EBPα pathway, BMP2/4 activates SMAD1/5/8-P through its receptors BMPR1A and BMPR2, which then form a complex with SMAD4, promoting the expression of C/EBPβ and PPARγ. The activation of C/EBPα depends on the early regulation of C/EBPβ, and through its interaction with PPARγ, it promotes fat accumulation. In the NF-κB pathway, TLR4 activates the MYD88 signaling pathway, further activating the IKK complex, leading to the degradation of IκB, releasing NF-κB into the nucleus. In the nucleus, NF-κB activates inflammatory genes such as TNF-α, IL-6, and IL-1β, which, through negative feedback, inhibit the expression of PPARγ and C/EBPα, indirectly suppressing adipogenesis. This figure created with BioGDP.com (Jiang et al., 2025).

Following IκBα degradation, the NF-κB heterodimer (typically composed of RelA/p65 and p50 subunits) is released and translocated into the nucleus, where it initiates the transcription of numerous pro-inflammatory genes (Fitzgerald and Kagan, 2020). For instance, infection with enterotoxigenic *Escherichia coli* K88 in weaned piglets activates TLR4/NF-κB signaling in the jejunum, triggering a robust inflammatory response. In an LPS-challenging model, microbiota-derived LPS directly activates this pathway, which was evidenced by increased phosphorylation of IκBα and NF-κB p65 in intestinal tissue and a marked upregulation of pro-inflammatory cytokines (Wang et al., 2019). Similarly, in a murine endotoxemia model, LPS administration induces a systemic inflammatory response characterized by TLR4/MyD88/NF-κB pathway activation in multiple organs. Probiotic supplementation can mitigate this systemic inflammation by downregulating this signaling cascade (Srivastava and Mohanty, 2025). For example, supplementation with *L. rhamnosus* has been shown to inhibit TLR4 expression and attenuate inflammation (Li et al., 2012). Other probiotics such as *C. butyricum* and *Enterococcus faecalis* similarly downregulate TLR4, MyD88, and NF-κB, reduce pro-inflammatory cytokine levels, and restore gut health, thereby indirectly normalizing lipid metabolism (Wang et al., 2019). Overall, microbiota-mediated modulation of the NF-κB pathway extends beyond inflammation control and includes effects on adipocyte apoptosis and functional regulation. Future research should aim to identify key microbial strains and metabolites that influence this pathway and explore novel regulatory mechanisms, such as microRNA-mediated interactions, to develop precise therapeutic strategies. Figure 1 displays the mechanism of gut microbiota regulating fat deposition.

### 4.6 Synthesis: an integrated network view of microbial metabolites in regulating fat deposition

A diverse array of microbial metabolites, including SCFAs, bile acids, tryptophan derivatives, BCAAs, and detrimental molecules like LPS, does not function independently, but forms a complex signaling network that centrally orchestrates host energy metabolism and lipid storage (Heng et al., 2020; Liu Y. et al., 2023). This network operates through three interconnected tiers.

First, the AMPK/mTOR system serves as the core hub for energy sensing and metabolic homeostasis. Microbial metabolites, such as butyrate, acetate, and BCAAs directly modulate AMPK and mTOR, thereby influencing metabolic programs (den Besten et al., 2015; Yoneshiro et al., 2019). AMPK activation promotes catabolic processes (e.g., fatty acid oxidation and lipolysis), whereas mTOR integrates nutrient signals to regulate anabolism and adipocyte differentiation. The integration of these microbial signals is therefore crucial for determining the host’s energy balance. Second, the PPARγ/C/EBPα axis acts as the transcriptional master regulator of adipogenesis. PPARγ and C/EBPα are master transcription factors driving adipogenesis and represent a convergence point for numerous microbial signals. For example, SCFAs, secondary bile acids (via FXR/TGR5), and tryptophan metabolites can inhibit the PPARγ/C/EBPα pathway, thereby preventing terminal adipocyte differentiation (Soundharrajan et al., 2020; Zhang et al., 2020). In contrast, gut dysbiosis and harmful metabolites like LPS can promote inflammation and thereby indirectly stimulating adipogenesis. Additionally, key developmental pathways such as Wnt/β-catenin and TGF-β/SMAD3 are modulated by metabolites like bile acids and butyrate, which further fine-tune fat production (Mouries et al., 2019; Chen et al., 2020). Third, epigenetic reprogramming functions as a chromatin-level interface and links the gut environment to long-term metabolic regulation (Lin et al., 2024). Metabolites including SCFAs (as HDAC inhibitors), bile acids, and tryptophan-derived indole derivatives (as AHR ligands) directly shape the host epigenome by altering histone acetylation, histone methylation, and DNA methylation patterns (Luu et al., 2019; Nshanian et al., 2025). These epigenetic changes can stably activate or silence key genes within the AMPK/mTOR and PPARγ/C/EBP*α* networks over the long term, thereby providing a molecular “memory” that influences susceptibility to obesity and metabolic diseases (Yang L. et al., 2025).

Critically, these tiers are highly interconnected and form an integrated regulatory circuit. For instance, SCFAs not only activate AMPK, but also inhibit HDACs to epigenetically activate fatty acid oxidation genes and suppress PPARγ signaling (Luu et al., 2019; Nshanian et al., 2025). Similarly, bile acids regulate gene expression via FXR signaling while concurrently shaping the epigenome (Soundharrajan et al., 2020; Zhang et al., 2020). This multi-tiered coordination ensures the precise and stable regulation of lipid metabolism. However, gut microbiota dysbiosis disrupts this homeostatic balance, leading to network dysfunction characterized by suppressed AMPK, overactive mTOR, dysregulated PPARγ-driven adipogenesis, and hyperactivation of the NF-κB pathway. Collectively, these alterations promote excessive fat deposition. Therefore, future therapeutic strategies should aim to restore the integrity of the entire host-microbiota regulatory network, rather than targeting isolated metabolites or pathways.

## 5 Translational applications

With growing insights into the mechanisms by which the gut microbiota influences obesity and metabolic diseases, microecology-based intervention strategies have transitioned from fundamental research into clinical and agricultural practice. Currently, five primary categories of microbial interventions are recognized: probiotics, prebiotics, synbiotics, postbiotics, and FMT. These strategies exhibit promising potential for improving metabolic health and regulating fat deposition in both humans and livestock.

### 5.1 Probiotics

Probiotics are defined as live microorganisms that, when administered in adequate amounts, confer health benefits to the host. As the critical role of gut microbiota in obesity pathogenesis becomes increasingly evident, probiotics have emerged as a key tool for modulating intestinal microecology. They hold broad promise for applications in weight management, metabolic regulation, and the optimization of animal production. Currently, over 60% of clinical trials involving probiotics report beneficial effects on weight control (Vallianou et al., 2020). Multiple randomized controlled trials have confirmed that specific probiotic strains, such as *L. gasseri* SBT2055 and *Bifidobacterium breve*, can effectively reduce body weight, body fat, and BMI by modulating host metabolism (Kadooka et al., 2010; Kondo et al., 2010). Notably, *L. gasseri* BNR17 has been approved in South Korea population as a functional food ingredient with lipid-lowering properties (Jung et al., 2013).

The efficacy of probiotics is mediated through multiple mechanisms. Probiotics can enhance intestinal barrier integrity, regulate immune responses, reduce systemic inflammation, and modulate lipid synthesis and energy absorption pathways. This is achieved both by producing beneficial microbial metabolites and by regulating key signaling pathways involved in lipid metabolism. These benefits have been consistently observed in animal models. For instance, supplementation with *Lactobacillus* or *Bifidobacterium* species in mice reduces fat accumulation, lowers circulating endotoxin levels, and suppresses inflammatory signaling (Chelakkot et al., 2018; Lee et al., 2025). Similarly, *Lactiplantibacillus plantarum* alleviates metabolic disorders by reshaping the gut microbiota, increasing SCFA production, and modulating adipose tissue and liver transcription via the PPAR and PI3K/Akt pathways, respectively (Gao et al., 2025). Furthermore, *Roseburia hominis* exerts its anti-obesity effects primarily through upregulation of the SIRT1/mTOR signaling pathway, as demonstrated by both its live bacteria and its culture medium. This mechanism is considered the primary pathway, with an additional contribution from reduced dietary energy absorption (Huang et al., 2025). Among emerging probiotics, *A. muciniphila* has garnered significant attention. Its abundance is negatively correlated with obesity, type 2 diabetes, and hypertension, positioning it as a promising next generation “candidate probiotic” (Cani and Everard, 2014). Pasteurized *A. muciniphila* has been shown to ameliorate obesity through multiple mechanisms including enhancing intestinal barrier integrity by upregulating *ZO-1* and *Occludin*, enriching SCFA-producing bacteria, increasing circulating SCFA and GLP-1 levels, and regulating key metabolic pathways via upregulation of AMPK/PPAR-α and downregulation of PPARγ signaling (Yang L. et al., 2025).

In livestock production, the application of probiotics has advanced from experimental trials to routine use, particularly in swine farming. Multi-strain formulations, such as combinations of *Lactobacillus* and *Bacillus*, have been shown to promote growth, enhance feed conversion efficiency, reduce diarrhea, and inhibit colonization by pathogenic bacteria such as enterotoxigenic *Escherichia coli* K88 (Guerra-Ordaz et al., 2014). Additionally, probiotics like *L. rhamnosus*, *L. plantarum*, and *Bacillus* have been reported to improve intestinal immunity and overall health status in pigs.

In general, probiotics consistently demonstrate anti-obesity and metabolic regulatory effects in both human and animal studies. These benefits are achieved by remodeling the gut microbiota, suppressing metabolic inflammation, and enhancing intestinal barrier function. Importantly, recent studies have elucidated the underlying molecular mechanisms, including the regulation of key signaling pathways (e.g., PPAR, AMPK, PI3K-Akt, mTOR) and key microbial metabolites (e.g., SCFAs, bile acids), which directly fine-tune host lipid metabolism and energy homeostasis. However, challenges remain, including limited mechanistic clarity for individual strains, a lack of standardized dosing protocols and intervention durations, and variable host-specific responses. Future research should focus on strain-level screening, mechanistic validation, and precision nutrition strategies to support the effective translation of probiotics into clinical and industrial applications.

### 5.2 Prebiotics

Prebiotics are non-digestible dietary components, such as polysaccharides, oligosaccharides, and certain fibers, that beneficially affect host health by selectively stimulating the growth and activity of beneficial gut microorganisms, particularly *Lactobacillus* and *Bifidobacterium* (Ji et al., 2023). These compounds are not directly absorbed or metabolized by the host, but instead act through microbiota-mediated mechanisms.

The efficacy of prebiotics is largely mediated by their capacity to reshape the gut microbiota and its metabolic output. For example, polysaccharides derived from marine algae, e.g., purple algae and reticular moss, increase the abundance of SCFA-producing bacteria and directly activate the AMPK/ACC signaling pathway. This promotes lipolysis and fatty acid oxidation while suppressing PPARγ, collectively contributing to a pronounced anti-obesity effect (Zheng M. et al., 2024; Zheng W. et al., 2024; Nohesara et al., 2025). Similarly, raspberry polysaccharides and the isoflavone formononetin enrich butyrate-producing bacteria, modulate hepatic miRNA expression, and influence inflammatory markers, thereby alleviating metabolic disorders (Nohesara et al., 2025). These findings highlight the presence of a microbiota-metabolite-epigenetics axis.

Furthermore, prebiotics help alleviate metabolic inflammation by reinforcing intestinal barrier integrity and inhibiting pro-inflammatory signaling. For instance, fucoidan and arctigenin enhance the gut barrier, elevate SCFA levels, and concurrently suppress the MAPK/NF-κB and TLR4/NF-κB pathways, thereby ameliorating obesity-associated pathologies (Wang N. et al., 2024; Zheng W. et al., 2024). Specifically, arctigenin promotes SCFA-producing bacterial populations, which increases SCFA levels, activates the GPR41/43 pathway, and inhibits HDAC3. These actions collectively maintain intestinal immune homeostasis and activate the hepatic GPR/AMPK axis, ultimately reducing lipid accumulation (Wang N. et al., 2024).

In human studies, diets enriched with prebiotics have been strongly associated with reductions in body weight and fat mass, particularly in overweight or obese individuals (Panichsillaphakit et al., 2021). In animal production, prebiotics have also been applied due to their regulatory effects on fat deposition and production performance. For example, combined supplementation with maternal-source probiotics and synbiotics significantly altered plasma metabolic indices in piglets, including reductions in cholinesterase, blood urea nitrogen, and glucose, while also modulating lipid-related parameters such as low-density lipoprotein cholesterol (LDL-C), total cholesterol, and triglycerides across different developmental stages (Zhu et al., 2022). These findings provide strong evidence for the application of prebiotics in improving lipid profiles.

Overall, prebiotics are natural, low-toxicity, and multi-faceted metabolic modulators with significant potential for improving host metabolism and body weight regulation. Their mechanisms are multifactorial. It not only selectively stimulates beneficial gut microbes but also activates host metabolic pathways (e.g., AMPK, PPARs) and facilitates epigenetic modifications (e.g., HDAC inhibition, miRNA regulation), largely through microbial metabolites such as SCFAs. Future research should focus on elucidating the structure–function relationships of specific prebiotics and optimizing their targeted applications in clinical and animal nutrition.

### 5.3 Synbiotics

Synbiotics are synergistic formulations combining probiotics and prebiotics based on the principle of functional complementarity. They aim to enhance the survival, colonization, and metabolic activity of beneficial microorganisms, thereby maintaining intestinal microecological balance and improving host health. Compared with probiotics or prebiotics administered individually, synbiotics offer improved probiotic viability in food or feed matrices and facilitate selective fermentation of prebiotics to expand target microbial populations. However, the efficacy of synbiotics is highly dependent on the compatibility between selected microbial strains and prebiotic substrates. This interaction is often complex, exhibiting synergistic, additive, or even antagonistic effects, which contribute to the diverse yet not fully understood potential of synbiotics in modulating the gut microbiota (Chen et al., 2023).

In recent years, synbiotics have gained attention as a promising strategy for addressing obesity and its associated metabolic disorders. Evidence suggests that synbiotics may confer greater metabolic benefits than probiotics or prebiotics alone. For example, a novel synbiotic comprising *Lactiplantibacillus plantarum* LLY-606 and galacto-oligosaccharides (GOS) was found to reduce visceral fat and alleviate obesity. A key mechanism involves elevating serum arginine levels, which in turn activates the AMPK signaling pathway to improve lipid metabolism. Causality was established through experiments which showed that inhibiting arginine synthesis or knocking out the bacterial argininosuccinate synthetase 1 (*Ass1*) gene abrogates these benefits (Shi et al., 2025). Similarly, a synbiotic combination of *Bifidobacterium*, *Lactobacillus*, *Lactococcus*, *Bacillus*, and omega-3 fatty acids significantly inhibited hepatic fat accumulation and reduced circulating lipid levels (Kobyliak et al., 2017).

The metabolic effects of synbiotics are closely linked to their capacity to modulate microbial metabolites. In diet-induced obese mice, synbiotic intervention restored HFD-induced declines in cecal SCFAs and reduced the expanded total bile acid pool, indicating comprehensive restoration of microbial metabolic functions (Ke et al., 2019). Another study showed that a synbiotic combining *C. butyricum* with corn gluten reduced pathogenic bacterial abundance, enhanced SCFA-producing microbes, and increased acetate and isopentanoate concentrations (Zhang et al., 2018). Similarly, *L. paracasei* N1115 plus oligofructose improved NAFLD phenotypes and downregulated TLR4 and NF-κB pathways (Yao et al., 2019). At the host signaling level, synbiotic lowered serum and hepatic LPS levels, thereby alleviating metabolic endotoxemia and inhibiting the hepatic TLR4/NF-κB pathway. Concurrently, it upregulated colonic gene expression of *GLP-1*, *GPR41*, and *GPR43* that connected reduced inflammation to improved glucose metabolism (Kang et al., 2023). Through these multi-targeted, synergistic mechanisms, synbiotics can reshape the gut microbiota, enhance beneficial metabolite production, restore intestinal barrier integrity, and mitigate inflammatory responses, collectively supporting their potential in preventing and managing obesity and metabolic diseases.

The efficacy of synbiotics is influenced by numerous factors, including formulation design, host genetics, and environmental conditions. The superior therapeutic effects observed with certain synbiotics likely stem from their ability to act synergistically by enriching key microbial communities, regulating critical metabolites such as short-chain fatty acids, bile acids, and arginine, and consequently coordinating host metabolic pathways including AMPK, NF-κB, and PPARs, and modulating tissue functions such as fat browning and hepatic lipid metabolism (Ke et al., 2019; Kang et al., 2023; Shi et al., 2025). Future research should prioritize mechanistic studies, formulation optimization, and the development of personalized intervention strategies to support the effective clinical translation and industrial application of synbiotics.

### 5.4 Postbiotics

Postbiotics are defined as functional substances composed of inanimate microorganisms or their cellular components that confer health benefits to the host. Due to their inherent stability, safety, and absence of infection risk, postbiotics have become an emerging focus in the field of microecological interventions.

In the context of metabolic regulation, postbiotics modulate fat deposition and systemic metabolic homeostasis via diverse signaling pathways. For example, extracellular polysaccharides derived from *L. plantarum* L-14 activate the TLR2–AMPK pathway, thereby inhibiting preadipocyte differentiation and reducing fat accumulation and dyslipidemia (Lee et al., 2021). Similarly, long-chain polyphosphates produced by *Lactobacillus johnsonii* alleviate intestinal inflammation and enhance epithelial barrier integrity through activation of the ERK signaling pathway (Isozaki et al., 2021). Other bacterial structural components, such as muramyl dipeptide (MDP), a cytosolic peptidoglycan fragment, have demonstrated anti-inflammatory and insulin-sensitizing properties via the NOD2–IRF4 signaling pathway. Interestingly, while MDP binding to NOD2 exerts beneficial effects, its interaction with NOD1 may exacerbate metabolic disturbances, emphasizing the receptor-specific nature of postbiotic actions (Cavallari et al., 2020).

Among microbial metabolites, butyrate represents the most extensively studied postbiotic molecule. It has been shown to strengthen intestinal barrier function by activating GPR43, upregulating NLRC3 expression via the TRAF6 axis, and increasing tight junction protein levels (e.g., ZO-1) (Cheng et al., 2018). In murine models of NAFLD, butyrate supplementation upregulated ZO-1 expression in the small intestine, lowered circulating endotoxin levels, and alleviated systemic metabolic inflammation associated with gut barrier dysfunction (Ye et al., 2018). In livestock production, postbiotics have also demonstrated potential for improving animal health and performance. For instance, dietary supplementation with heat-killed *Lactobacillus sali*var*ius* 189 improved gut microbial composition in pigs by reducing the abundance of *Prevotella* and increasing *Parabacteroides*, correlating with reduced fat accumulation (Ryu et al., 2022). Similarly, supplementation with *L. rhamnosus* (heat-treated at 80 °C for 30 min, 1 × 10^9^ CFU/g) improved piglet growth performance, feed conversion ratio, and dry matter digestibility, while also reducing the incidence of diarrhea and serum levels of TNF-*α*, TGF-β1, and cortisol (Kang et al., 2021).

As a cutting-edge strategy in microbiota-based interventions, postbiotics offer a promising avenue for metabolic disease prevention, gut barrier restoration, and livestock productivity enhancement, owing to their controlled composition, enhanced safety profile, and immunomodulatory potential. By activating specific host receptors, modulating immune and metabolic signaling, and improving barrier and endocrine function, postbiotics expand the functional spectrum of microecological therapies. Future research should prioritize the functional characterization of active compounds, elucidation of receptor-mediated mechanisms, and development of personalized, context-specific applications to enable their efficient translation into human health management and animal agriculture.

### 5.5 FMT

FMT is a microecological intervention strategy that aims to restore gut microbiota composition and function in a recipient by transferring fecal microbiota from a healthy donor. Initially developed for the treatment of recurrent *Clostridioides difficile* infection, with consistently high clinical efficacy, FMT has since gained attention for its potential in addressing obesity, metabolic syndrome, and related conditions. Recent advances in gut microbiome research have revealed the strong association between gut microbiota and metabolic diseases, which has driven interest in expanding the therapeutic scope of FMT. Although preliminary clinical studies suggest that FMT can improve insulin sensitivity, the overall efficacy of FMT in metabolic interventions remains inconclusive, likely due to limitations such as small sample sizes, short intervention durations, and donor–recipient microbiota heterogeneity (Kootte et al., 2017).

FMT improves host metabolism by reshaping the gut microbial ecosystem and reactivating key metabolic pathways, including SCFAs and bile acid metabolism. FMT promotes the colonization of beneficial taxa, such as butyrate-producing bacteria (*Faecalibacterium*, *Roseburia intestinalis*), species with BSH activity, and next-generation probiotics such as *A. muciniphila* (Dao et al., 2016). These microbial shifts enhance GLP-1 secretion and improve insulin sensitivity. Supplementation with *A. muciniphila* alone has been shown to improve insulin sensitivity and reduce total cholesterol and fat mass (Depommier et al., 2019). In livestock, FMT has also shown promising effects on fat deposition. The gut microbial composition of pigs is closely linked to their adiposity profiles. “Fat-type” breeds such as Rongchang and Jinhua pigs have a significantly higher Firmicutes/Bacteroidetes ratio than lean breeds like Yorkshire and Landrace pigs (Yang et al., 2018). Yan et al. (2016) demonstrated that mice receiving FMT from Rongchang or Yorkshire pigs exhibited increased fat deposition, confirming that gut microbiota can mediate cross-species transmission of obesity-associated traits.

**Figure 2:**
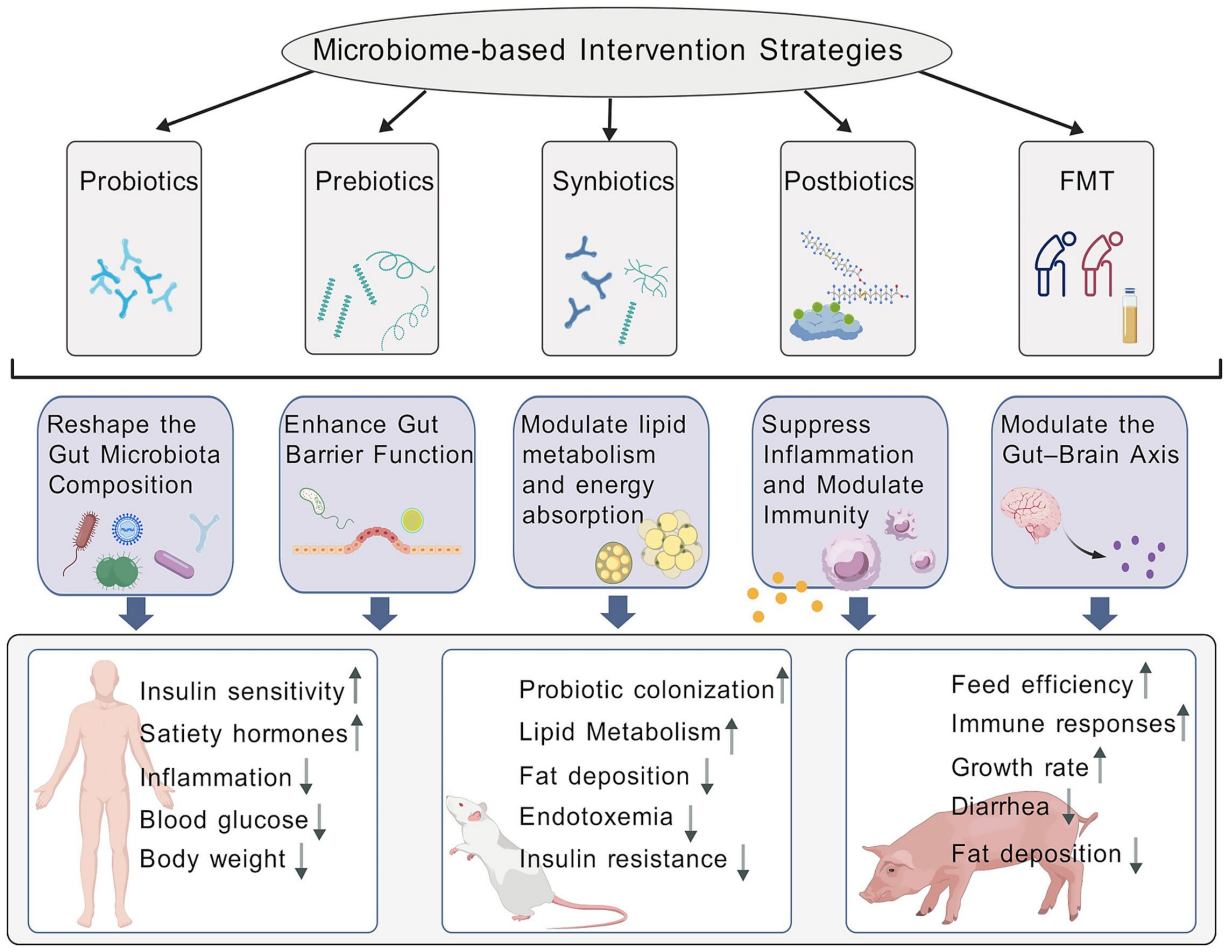
Microbiome-based intervention strategies and their effects on gut health, metabolism, and systemic function. This figure created with BioGDP.com (Jiang et al., 2025).

Although FMT has a promising future in metabolic disease intervention, its clinical application still faces multiple challenges, including: firstly, inconsistent donor selection criteria; secondly, a lack of standardization of FMT preparation and preservation procedures; and thirdly, differences in the background of the recipient’s microbiota affecting implantation efficacy. Studies have shown that the diversity of the recipient’s own microbiota and the structure of the core microbiota have a significant impact on the effectiveness of FMT (Danne et al., 2021). Therefore, individualized FMT strategies should be developed to optimize the colonization effect. In the field of animal husbandry, FMT can be used as a new strategy to improve production performance. For example, transplanting lean pig colonies to fat pigs is expected to reduce fat deposition and increase lean meat percentage (Yan et al., 2016). However, key issues such as long-term stability and safety of the colonies still need to be addressed to realize this goal and ensure their sustainable application. Figure 2 displays the microbiome-based intervention strategies and their effects on gut health, metabolism, and systemic function.

## 6 Challenges and future directions

The pivotal role of the gut microbiota in regulating adipose tissue deposition and metabolic disorders has garnered extensive attention, catalyzing the development of diverse intervention strategies, including probiotics, prebiotics, and FMT. Despite these advances, significant challenges persist across fundamental mechanistic research, clinical translation, and the integration of emerging technologies, necessitating systematic breakthroughs to advance the field.

### 6.1 Bottlenecks in mechanism investigation

#### 6.1.1 Challenges in establishing causality

Discerning causal relationships between the gut microbiota and host metabolic phenotypes remains a central focus in microbial ecology. However, the intrinsic complexity of microbial communities coupled with multifactorial host and environmental influences has largely confined current research to correlative associations, impeding clear differentiation of whether microbial alterations are causative drivers or consequential markers of disease (Cani et al., 2021). Although interventional approaches, such as FMT and GF animal models, have provided preliminary evidence for causality, these methodologies are technically demanding, costly, and prone to confounding effects due to variability in individual baseline microbiota. Notably, identical microbial interventions often yield heterogeneous responses among recipients, with pronounced inter-individual differences observed in probiotic efficacy and FMT outcomes. This biological heterogeneity severely constrains reproducibility and generalizability, underscoring the urgent need for the development of standardized, quantitative frameworks to robustly validate causality and thereby enhance mechanistic insights.

#### 6.1.2 Limitations imposed by species differences on translational validity

Mechanistic investigations into host-microbiota interactions predominantly rely on murine models. Nonetheless, fundamental interspecies disparities in physiological metabolism, immune system dynamics, and gut microbial composition critically limit the extrapolation of findings from mice to humans. For instance, discrepancies exist in the relative abundance of dominant bacterial phyla, colonization capacity of human-derived pathogens, and immune pathway activation patterns between murine and human gut ecosystems (Ley et al., 2008), diminishing the translational relevance of mouse-derived data.

Recently, porcine models have gained traction due to their closer resemblance to human anatomy and metabolic physiology. However, comprehensive functional annotation of the porcine microbiome and elucidation of its metabolic interaction mechanisms remain underdeveloped. Furthermore, cross-species validation of microbiota-mediated mechanisms is lacking. Future research should emphasize a “multi-model validation” strategy, integrating rodent, large animal, and advanced *in vitro* organoid models, to enhance mechanistic understanding and facilitate more reliable translation of preclinical findings to human contexts.

### 6.2 Challenges in translational application

#### 6.2.1 Stability and persistence of microbiota-based interventions

Although interventions such as probiotics, prebiotics, postbiotics, and FMT have demonstrated promising effects in mitigating obesity and metabolic disorders in short-term studies, comprehensive and systematic evaluations of their long-term efficacy, safety, and holistic impacts on host microbial ecology remain insufficient. This issue is particularly critical in the context of animal husbandry, where probiotic and prebiotic supplementation has emerged as a novel approach to enhance animal performance and meat quality. However, several key challenges impede their widespread application (Swanson et al., 2025).

First, formulation stability is paramount; probiotic strains must withstand gastric acid, bile salts, digestive enzymes, and thermal stresses encountered during feed processing without significant loss of viability. Second, storage and feeding conditions critically influence microbial viability, as exposure to elevated temperature and humidity often diminishes strain activity, compromising intervention efficacy. Third, interactions with feed components may lead to antagonistic or destabilizing effects between microbial formulations and other nutritional ingredients, undermining functional outcomes. Fourth, persistence of functional expression remains to be fully elucidated, including the delivery efficiency, absorption kinetics, and specific target engagement of bioactive compounds such as prebiotics and postbiotics within the host.

Addressing these challenges necessitates the development of more robust, storage-stable formulations with precisely targeted release mechanisms, alongside rigorous evaluation of their synergistic compatibility with complex feed matrices. Such advances are critical to facilitating the effective and sustainable implementation of microecological interventions in animal production systems.

#### 6.2.2 Increasing demand for individualized modulation

In human metabolic regulation, the demand for individualized microbiota-based interventions is growing. Due to significant inter-individual differences in gut microbial composition, metabolic activity, and host genetic background, responses to the same intervention can vary greatly. For instance, some individuals respond well to specific probiotic strains, while others exhibit no apparent effect (Ejtahed et al., 2017).

Personalized microecological interventions require the integration of multi-omics data, encompassing microbiota configuration, metabolomic features, host genotypes, and dietary behaviors (Rouskas et al., 2025). However, such personalized modulation strategies are still in the early stages of exploration and face challenges including high data acquisition costs, complex model construction, and long clinical validation cycles. Establishing actionable predictive models that link “microbiota–host–intervention outcomes” represents a critical direction for future research in precision nutrition and precision medicine.

### 6.3 Breakthroughs driven by cutting-edge technologies

#### 6.3.1 Organoid models for *in vitro* mechanism simulation

Organoid technology, a three-dimensional cell culture system that has emerged in recent years, effectively recapitulates the structural and functional characteristics of native tissues and organs. The establishment of co-culture systems combining intestinal organoids with microorganisms offers novel insights into the mechanistic basis of host–microbiota interactions. Compared to traditional two-dimensional cell lines, organoid models provide superior physiological relevance, enabling dynamic observation of microbial or metabolite-mediated regulatory effects on intestinal epithelial and immune cells (Zheng L. et al., 2024). Looking ahead, organoid platforms are poised to become indispensable tools for high-throughput screening of novel microecological therapeutics and functional validation.

#### 6.3.2 Single-cell technologies enhance functional resolution

While metagenomics and metabolomics afford a broad perspective on microbial community functions, they remain limited in resolving functions at the level of individual bacterial cells and their precise interactions with host cells. Single-cell sequencing technologies facilitate high-resolution characterization of microbial functional states, gene expression profiles, and microbe–host cell interactions (Madhu et al., 2023). Utilizing single-cell transcriptomics, researchers can delineate cell-type-specific responses of intestinal epithelial cells or immune cells to microbial stimuli and identify core signaling pathways regulating lipid metabolism. Additionally, these approaches enable the discovery of novel regulatory targets, including transcription factors and regulatory elements modulated by specific bacterial strains.

#### 6.3.3 Artificial intelligence for constructing “microbe-host” interaction networks

Artificial intelligence (AI) has demonstrated a remarkable capacity for analyzing multi-omics datasets, detecting complex patterns, and constructing predictive models. Leveraging machine learning and deep learning frameworks, AI can integrate metagenomic, transcriptomic, metabolomic, and clinical phenotypic data to forecast host responses to diverse microbial interventions. For example, AI models have been developed to predict individual response variability following probiotic administration and to simulate metabolic trajectories post-FMT (Patil et al., 2025). Shortly, AI is expected to be pivotal in transforming gut microbiology research from descriptive characterization to predictive modeling, thereby providing robust algorithmic support for precision nutrition, precision medicine, and precision animal breeding.

#### 6.3.4 System modeling and metabolite-receptor mapping for deciphering causal mechanisms

Leveraging the predictive power of artificial intelligence (AI), system modeling has emerged as a critical research direction and a major trend for future development. Methods such as constraint-based reconstruction and analysis (COBRA) and genome-scale metabolic models (GEMs) provide a powerful computational platform for quantitatively simulating the interactions between the gut microbiota and the host’s metabolic network (Li G. H. et al., 2025; Taubenheim et al., 2025). These modeling approaches extend beyond traditional correlation analyses. They can predict metabolic fluxes, identify key metabolic pathways in conditions like obesity and inflammatory bowel disease (IBD), and screen for potential therapeutic metabolites or dietary interventions through in silico simulations (Taubenheim et al., 2025). The integration of metabolic models with metabolite-receptor interaction maps represents a promising strategy to bridge the gap between microbial metabolism and host physiology. This strategy functions by linking specific microbial metabolites to their cognate host receptors. For instance, it elucidates how metabolites, including short-chain fatty acids and tryptophan derivatives, engage receptors such as the aryl hydrocarbon receptor, GPCRs, and the pregnane X receptor, thereby establishing causal relationships between the microbiota and the regulation of host immunity and metabolism (Koduru et al., 2022). Ultimately, the combination of system modeling and receptor mapping is pivotal for translating complex microbiome data into targeted microbial therapies with well-defined mechanisms of action.

## 7 Discussion

As a critical regulator of metabolic homeostasis, the gut microbiota holds substantial potential for preventing and treating obesity and related metabolic disorders. Nonetheless, significant challenges remain, including difficulties in establishing causality in mechanistic studies, interspecies differences limiting translational applicability, insufficient long-term evaluation of microbiota-targeted interventions, and the pressing need for individualized therapeutic strategies. The advent of advanced technologies such as organoid culture, single-cell sequencing, and AI-driven modeling is progressively overcoming the constraints inherent to traditional methodologies and ushering in a new era characterized by high-throughput, high-precision, and personalized microbiome regulation.

Future studies should include elucidating the specific roles of microbial metabolites in host lipid metabolism, developing cross-species, high-fidelity platforms for causal validation, optimizing the stability and targeting efficiency of microbial interventions, constructing multi-omics-guided personalized intervention frameworks, and accelerating the translation of scientific discoveries into clinical and agricultural applications. These endeavors will facilitate the establishment of scientifically rigorous, systematic, and sustainable approaches for precise prevention and management of obesity and metabolic diseases, as well as for enhancing the economic performance of animal husbandry.

## References

[ref1] Agudelo L. Z. Ferreira D. M. S. Cervenka I. Bryzgalova G. Dadvar S. Jannig P. R. . (2018). Kynurenic acid and Gpr35 regulate adipose tissue energy homeostasis and inflammation. Cell Metab. 27, 378–392.e375. doi: 10.1016/j.cmet.2018.01.004, 29414686

[ref2] Agus A. Clément K. Sokol H. (2021). Gut microbiota-derived metabolites as central regulators in metabolic disorders. Gut 70, 1174–1182. doi: 10.1136/gutjnl-2020-323071, 33272977 PMC8108286

[ref3] Arany Z. Neinast M. (2018). Branched chain amino acids in metabolic disease. Curr. Diab. Rep. 18:76. doi: 10.1007/s11892-018-1048-7 30112615

[ref4] Bäckhed F. Ding H. Wang T. Hooper L. V. Koh G. Y. Nagy A. . (2004). The gut microbiota as an environmental factor that regulates fat storage. Proc. Natl. Acad. Sci. USA 101, 15718–15723. doi: 10.1073/pnas.0407076101, 15505215 PMC524219

[ref5] Bastings J. Venema K. Blaak E. E. Adam T. C. (2023). Influence of the gut microbiota on satiety signaling. Trends Endocrinol. Metab. 34, 243–255. doi: 10.1016/j.tem.2023.02.003, 36870872

[ref6] Behera J. Ison J. Voor M. J. Tyagi N. (2021). Probiotics stimulate bone formation in obese mice via histone methylations. Theranostics 11, 8605–8623. doi: 10.7150/thno.63749, 34373761 PMC8344023

[ref7] Cani P. D. Amar J. Iglesias M. A. Poggi M. Knauf C. Bastelica D. . (2007). Metabolic endotoxemia initiates obesity and insulin resistance. Diabetes 56, 1761–1772. doi: 10.2337/db06-1491, 17456850

[ref8] Cani P. D. Bibiloni R. Knauf C. Waget A. Neyrinck A. M. Delzenne N. M. . (2008). Changes in gut microbiota control metabolic endotoxemia-induced inflammation in high-fat diet-induced obesity and diabetes in mice. Diabetes 57, 1470–1481. doi: 10.2337/db07-1403, 18305141

[ref9] Cani P. D. Everard A. (2014). *Akkermansia muciniphila*: a novel target controlling obesity, type 2 diabetes and inflammation? Med. Sci. (Paris) 30, 125–127. doi: 10.1051/medsci/20143002003, 24572104

[ref10] Cani P. D. Moens de Hase E. Van Hul M. (2021). Gut microbiota and host metabolism: from proof of concept to therapeutic intervention. Microorganisms 9:1302. doi: 10.3390/microorganisms9061302, 34203876 PMC8232674

[ref11] Caradonna E. Abate F. Schiano E. Paparella F. Ferrara F. Vanoli E. . (2025). Trimethylamine-N-oxide (TMAO) as a rising-star metabolite: implications for human health. Meta 15:220. doi: 10.3390/metabo15040220, 40278349 PMC12029716

[ref12] Cavallari J. F. Barra N. G. Foley K. P. Lee A. Duggan B. M. Henriksbo B. D. . (2020). Postbiotics for NOD2 require nonhematopoietic RIPK2 to improve blood glucose and metabolic inflammation in mice. Am. J. Physiol. Endocrinol. Metab. 318, E579–e585. doi: 10.1152/ajpendo.00033.2020, 32101030

[ref13] Cemin H. S. Tokach M. D. Woodworth J. C. Dritz S. S. DeRouchey J. M. Goodband R. D. (2019). Branched-chain amino acid interactions in growing pig diets. Transl. Anim. Sci. 3, 1246–1253. doi: 10.1093/tas/txz087, 32704888 PMC7200481

[ref14] Chelakkot C. Choi Y. Kim D. K. Park H. T. Ghim J. Kwon Y. . (2018). *Akkermansia muciniphila*-derived extracellular vesicles influence gut permeability through the regulation of tight junctions. Exp. Mol. Med. 50:e450. doi: 10.1038/emm.2017.282, 29472701 PMC5903829

[ref15] Chen C. Fang S. Wei H. He M. Fu H. Xiong X. . (2021). *Prevotella copri* increases fat accumulation in pigs fed with formula diets. Microbiome 9:175. doi: 10.1186/s40168-021-01110-0 34419147 PMC8380364

[ref16] Chen D. Jin D. Huang S. Wu J. Xu M. Liu T. . (2020). *Clostridium butyricum*, a butyrate-producing probiotic, inhibits intestinal tumor development through modulating Wnt signaling and gut microbiota. Cancer Lett. 469, 456–467. doi: 10.1016/j.canlet.2019.11.019, 31734354

[ref17] Chen T. Wang J. Liu Z. Gao F. (2023). Effect of supplementation with probiotics or synbiotics on cardiovascular risk factors in patients with metabolic syndrome: a systematic review and meta-analysis of randomized clinical trials. Front. Endocrinol. (Lausanne) 14:1282699. doi: 10.3389/fendo.2023.1282699, 38260154 PMC10801034

[ref18] Cheng D. Xu J. H. Li J. Y. Wang S. Y. Wu T. F. Chen Q. K. . (2018). Butyrate ameliorated-NLRC3 protects the intestinal barrier in a GPR43-dependent manner. Exp. Cell Res. 368, 101–110. doi: 10.1016/j.yexcr.2018.04.018, 29689277

[ref19] Chiang J. Y. L. Ferrell J. M. (2019). Bile acids as metabolic regulators and nutrient sensors. Annu. Rev. Nutr. 39, 175–200. doi: 10.1146/annurev-nutr-082018-124344, 31018107 PMC6996089

[ref20] Collaborators G. A. B. (2025). Global, regional, and national prevalence of adult overweight and obesity, 1990-2021, with forecasts to 2050: a forecasting study for the global burden of disease study 2021. Lancet 405, 813–838. doi: 10.1016/S0140-6736(25)00355-1, 40049186 PMC11920007

[ref21] Collins S. L. Stine J. G. Bisanz J. E. Okafor C. D. Patterson A. D. (2023). Bile acids and the gut microbiota: metabolic interactions and impacts on disease. Nat. Rev. Microbiol. 21, 236–247. doi: 10.1038/s41579-022-00805-x, 36253479 PMC12536349

[ref22] Crovesy L. Ostrowski M. Ferreira D. Rosado E. L. Soares-Mota M. (2017). Effect of Lactobacillus on body weight and body fat in overweight subjects: a systematic review of randomized controlled clinical trials. Int. J. Obes. 41, 1607–1614. doi: 10.1038/ijo.2017.161, 28792488

[ref23] Danne C. Rolhion N. Sokol H. (2021). Recipient factors in faecal microbiota transplantation: one stool does not fit all. Nat. Rev. Gastroenterol. Hepatol. 18, 503–513. doi: 10.1038/s41575-021-00441-5, 33907321

[ref24] Dao M. C. Everard A. Aron-Wisnewsky J. Sokolovska N. Prifti E. Verger E. O. . (2016). Akkermansia muciniphila and improved metabolic health during a dietary intervention in obesity: relationship with gut microbiome richness and ecology. Gut 65, 426–436. doi: 10.1136/gutjnl-2014-308778, 26100928

[ref25] de la Oa V. Zazpe I. Ruiz-Canela M. (2020). Effect of branched-chain amino acid supplementation, dietary intake and circulating levels in cardiometabolic diseases: an updated review. Curr. Opin. Clin. Nutr. Metab. Care 23, 35–50. doi: 10.1097/MCO.0000000000000614, 31688095

[ref26] Dehghan P. Farhangi M. A. Nikniaz L. Nikniaz Z. Asghari-Jafarabadi M. (2020). Gut microbiota-derived metabolite trimethylamine N-oxide (TMAO) potentially increases the risk of obesity in adults: an exploratory systematic review and dose-response meta- analysis. Obes. Rev. 21:e12993. doi: 10.1111/obr.12993, 32017391

[ref27] Delbaere K. Roegiers I. Bron A. Durif C. Van de Wiele T. Blanquet-Diot S. . (2023). The small intestine: dining table of host-microbiota meetings. FEMS Microbiol. Rev. 47:fuad022. doi: 10.1093/femsre/fuad022, 37193669 PMC10246847

[ref28] den Besten G. Bleeker A. Gerding A. van Eunen K. Havinga R. van Dijk T. H. . (2015). Short-chain fatty acids protect against high-fat diet-induced obesity via a PPARγ-dependent switch from lipogenesis to fat oxidation. Diabetes 64, 2398–2408. doi: 10.2337/db14-1213, 25695945

[ref29] Depommier C. Everard A. Druart C. Plovier H. Van Hul M. Vieira-Silva S. . (2019). Supplementation with *Akkermansia muciniphila* in overweight and obese human volunteers: a proof-of-concept exploratory study. Nat. Med. 25, 1096–1103. doi: 10.1038/s41591-019-0495-2, 31263284 PMC6699990

[ref30] Devkota S. Wang Y. Musch M. W. Leone V. Fehlner-Peach H. Nadimpalli A. . (2012). Dietary-fat-induced taurocholic acid promotes pathobiont expansion and colitis in Il10−/− mice. Nature 487, 104–108. doi: 10.1038/nature11225, 22722865 PMC3393783

[ref98] Dong Z. Yang S. Tang C. Li D. Kan Y. Yao L. (2025). New insights into microbial bile salt hydrolases: from physiological roles to potential applications. Front Microbiol. 16:1513541. doi: 10.3389/fmicb.2025.1513541 40012771 PMC11860951

[ref31] Du J. Zhang P. Luo J. Shen L. Zhang S. Gu H. . (2021). Dietary betaine prevents obesity through gut microbiota-drived microRNA-378a family. Gut Microbes 13, 1–19. doi: 10.1080/19490976.2020.1862612, 33550882 PMC7889173

[ref32] Ejtahed H. S. Angoorani P. Soroush A. R. Hasani-Ranjbar S. Siadat S. D. Larijani B. (2020). Gut microbiota-derived metabolites in obesity: a systematic review. Biosci. Microbiota Food Health 39, 65–76. doi: 10.12938/bmfh.2019-026, 32775123 PMC7392910

[ref33] Ejtahed H. S. Hasani-Ranjbar S. Larijani B. (2017). Human microbiome as an approach to personalized medicine. Altern. Ther. Health Med. 23, 8–9, 28987073

[ref34] Eshleman E. M. Rice T. Potter C. Waddell A. Hashimoto-Hill S. Woo V. . (2024). Microbiota-derived butyrate restricts tuft cell differentiation via histone deacetylase 3 to modulate intestinal type 2 immunity. Immunity 57, 319–332.e316. doi: 10.1016/j.immuni.2024.01.002, 38295798 PMC10901458

[ref35] Fan Y. Qian H. Zhang M. Tao C. Li Z. Yan W. . (2023). Caloric restriction remodels the hepatic chromatin landscape and bile acid metabolism by modulating the gut microbiota. Genome Biol. 24:98. doi: 10.1186/s13059-023-02938-5 37122023 PMC10150505

[ref36] Fang X. Liu S. Muhammad B. Zheng M. Ge X. Xu Y. . (2024). Gut microbiota dysbiosis contributes to α-synuclein-related pathology associated with C/EBPβ/AEP signaling activation in a mouse model of Parkinson's disease. Neural Regen. Res. 19, 2081–2088. doi: 10.4103/1673-5374.391191, 38227539 PMC11040317

[ref37] Fitzgerald K. A. Kagan J. C. (2020). Toll-like receptors and the control of immunity. Cell 180, 1044–1066. doi: 10.1016/j.cell.2020.02.041, 32164908 PMC9358771

[ref38] Ganapathy T. Yuan J. Ho M. Y. Wu K. K. Hoque M. M. Wang B. . (2025). Adipocyte FMO3-derived TMAO induces WAT dysfunction and metabolic disorders by promoting inflammasome activation in ageing. Nat. Commun. 16:8873. doi: 10.1038/s41467-025-63905-1 41053195 PMC12501297

[ref39] Gao X. Liu X. Xu J. Xue C. Xue Y. Wang Y. (2014). Dietary trimethylamine N-oxide exacerbates impaired glucose tolerance in mice fed a high fat diet. J. Biosci. Bioeng. 118, 476–481. doi: 10.1016/j.jbiosc.2014.03.001, 24721123

[ref40] Gao Y. Zhu A. Li J. Liu H. Li X. Zhang H. (2025). Lactiplantibacillus plantarum attenuates diet-induced obesity and insulin resistance through gut microbiota-driven PPAR/PI3K-Axis modulation. Microb. Biotechnol. 18:e70227. doi: 10.1111/1751-7915.70227, 41159770 PMC12570210

[ref41] Garavaglia B. Vallino L. Ferraresi A. Esposito A. Salwa A. Vidoni C. . (2022). Butyrate inhibits colorectal Cancer cell proliferation through autophagy degradation of β-catenin regardless of APC and β-catenin mutational status. Biomedicine 10:1131. doi: 10.3390/biomedicines10051131, 35625868 PMC9138675

[ref42] Geng J. Ni Q. Sun W. Li L. Feng X. (2022). The links between gut microbiota and obesity and obesity related diseases. Biomed. Pharmacother. 147:112678. doi: 10.1016/j.biopha.2022.112678 35134709

[ref43] Ghaderi F. Sotoodehnejadnematalahi F. Hajebrahimi Z. Fateh A. Siadat S. D. (2022). Effects of active, inactive, and derivatives of *Akkermansia muciniphila* on the expression of the endocannabinoid system and PPARs genes. Sci. Rep. 12:10031. doi: 10.1038/s41598-022-13840-8 35705595 PMC9200819

[ref44] Goswami C. Iwasaki Y. Yada T. (2018). Short-chain fatty acids suppress food intake by activating vagal afferent neurons. J. Nutr. Biochem. 57, 130–135. doi: 10.1016/j.jnutbio.2018.03.009, 29702431

[ref45] Guerra-Ordaz A. A. González-Ortiz G. La Ragione R. M. Woodward M. J. Collins J. W. Pérez J. F. . (2014). Lactulose and *Lactobacillus plantarum*, a potential complementary synbiotic to control postweaning colibacillosis in piglets. Appl. Environ. Microbiol. 80, 4879–4886. doi: 10.1128/AEM.00770-14, 24907322 PMC4135760

[ref46] Guo W. Zhang Z. Li L. Liang X. Wu Y. Wang X. . (2022). Gut microbiota induces DNA methylation via SCFAs predisposing obesity-prone individuals to diabetes. Pharmacol. Res. 182:106355. doi: 10.1016/j.phrs.2022.106355, 35842183

[ref47] Hamer H. M. Jonkers D. Venema K. Vanhoutvin S. Troost F. J. Brummer R. J. (2008). Review article: the role of butyrate on colonic function. Aliment. Pharmacol. Ther. 27, 104–119. doi: 10.1111/j.1365-2036.2007.03562.x, 17973645

[ref48] He Y. Cai X. Liu H. Conde K. M. Xu P. Li Y. . (2021). 5-HT recruits distinct neurocircuits to inhibit hunger-driven and non-hunger-driven feeding. Mol. Psychiatry 26, 7211–7224. doi: 10.1038/s41380-021-01220-z, 34290371 PMC8776930

[ref49] Hemachandra S. Rathnayake S. N. Jayamaha A. A. Francis B. S. Welmillage D. Kaur D. N. . (2025). Fecal microbiota transplantation as an alternative method in the treatment of obesity. Cureus 17:e76858. doi: 10.7759/cureus.76858, 39901991 PMC11788455

[ref9001] Henao-Mejia J. Elinav E. Jin C. Hao L. Mehal W. Z. Strowig T. . (2012). Inflammasome-mediated dysbiosis regulates progression of NAFLD and obesity. Nature. 482, 179–185. doi: 10.1038/nature10809 22297845 PMC3276682

[ref50] Heng J. Wu Z. Tian M. Chen J. Song H. Chen F. . (2020). Excessive BCAA regulates fat metabolism partially through the modification of m(6)a RNA methylation in weanling piglets. Nutr. Metab. (Lond.) 17:10. doi: 10.1186/s12986-019-0424-x 31998401 PMC6979292

[ref51] Hogan K. A. Ravindran A. Podolsky M. A. Glick A. B. (2013). The TGFβ1 pathway is required for NFκB dependent gene expression in mouse keratinocytes. Cytokine 64, 652–659. doi: 10.1016/j.cyto.2013.09.004, 24075100 PMC3942663

[ref52] Hou G. Peng W. Wei L. Li R. Yuan Y. Huang X. . (2020). *Lactobacillus delbrueckii* interfere with bile acid enterohepatic circulation to regulate cholesterol metabolism of growing-finishing pigs via its bile salt hydrolase activity. Front. Nutr. 7:617676. doi: 10.3389/fnut.2020.617676, 33363199 PMC7759492

[ref53] Hu H. Li A. Shi C. Chen L. Zhao Z. Yin X. . (2024). Mulberry branch fiber improved lipid metabolism and egg yolk fatty acid composition of laying hens via the enterohepatic axis. Microbiome 12:73. doi: 10.1186/s40168-024-01788-y 38605412 PMC11010431

[ref54] Huang K. Cai S. Fu T. Zhu Q. Liu L. Yao Z. . (2024). Wnt10b regulates osteogenesis of adipose-derived stem cells through Wnt/β-catenin signalling pathway in osteoporosis. Cell Prolif. 57:e13522. doi: 10.1111/cpr.13522, 37340715 PMC10771102

[ref55] Huang W. Zhu W. Lin Y. Chan F. K. L. Xu Z. Ng S. C. (2025). *Roseburia hominis* improves host metabolism in diet-induced obesity. Gut Microbes 17:2467193. doi: 10.1080/19490976.2025.2467193 39976263 PMC11845086

[ref56] Isozaki S. Konishi H. Fujiya M. Tanaka H. Murakami Y. Kashima S. . (2021). Probiotic-derived polyphosphate accelerates intestinal epithelia wound healing through inducing platelet-derived mediators. Mediat. Inflamm. 2021:5582943. doi: 10.1155/2021/5582943 PMC8025129 33859537

[ref57] Ji J. Jin W. Liu S. J. Jiao Z. Li X. (2023). Probiotics, prebiotics, and postbiotics in health and disease. MedComm (2020) 4:e420. doi: 10.1002/mco2.420 37929014 PMC10625129

[ref58] Jiang S. Li H. Zhang L. Mu W. Zhang Y. Chen T. . (2025). Generic diagramming platform (GDP): a comprehensive database of high-quality biomedical graphics. Nucleic Acids Res. 53, D1670–d1676. doi: 10.1093/nar/gkae973, 39470721 PMC11701665

[ref59] Jiao A. Yu B. He J. Yu J. Zheng P. Luo Y. . (2020). Short chain fatty acids could prevent fat deposition in pigs via regulating related hormones and genes. Food Funct. 11, 1845–1855. doi: 10.1039/C9FO02585E, 32067021

[ref60] Jiao A. Yu B. He J. Yu J. Zheng P. Luo Y. . (2021). Sodium acetate, propionate, and butyrate reduce fat accumulation in mice via modulating appetite and relevant genes. Nutrition 87-88:111198. doi: 10.1016/j.nut.2021.111198 33761444

[ref61] Joyce S. A. MacSharry J. Casey P. G. Kinsella M. Murphy E. F. Shanahan F. . (2014). Regulation of host weight gain and lipid metabolism by bacterial bile acid modification in the gut. Proc. Natl. Acad. Sci. USA 111, 7421–7426. doi: 10.1073/pnas.1323599111, 24799697 PMC4034235

[ref62] Jung S. P. Lee K. M. Kang J. H. Yun S. I. Park H. O. Moon Y. . (2013). Effect of *Lactobacillus gasseri* BNR17 on overweight and obese adults: a randomized, double-blind clinical trial. Korean J. Fam. Med. 34, 80–89. doi: 10.4082/kjfm.2013.34.2.80, 23560206 PMC3611107

[ref63] Jyoti Dey P. (2025). Mechanisms and implications of the gut microbial modulation of intestinal metabolic processes. NPJ Metab. Health Dis. 3:24. doi: 10.1038/s44324-025-00066-1 40604123 PMC12441142

[ref64] Kadooka Y. Sato M. Imaizumi K. Ogawa A. Ikuyama K. Akai Y. . (2010). Regulation of abdominal adiposity by probiotics (*Lactobacillus gasseri* SBT2055) in adults with obese tendencies in a randomized controlled trial. Eur. J. Clin. Nutr. 64, 636–643. doi: 10.1038/ejcn.2010.19, 20216555

[ref65] Kang J. Lee J. J. Cho J. H. Choe J. Kyoung H. Kim S. H. . (2021). Effects of dietary inactivated probiotics on growth performance and immune responses of weaned pigs. J. Anim. Sci. Technol. 63, 520–530. doi: 10.5187/jast.2021.e44, 34189502 PMC8203999

[ref66] Kang Y. Ren P. Shen X. Kuang X. Yang X. Liu H. . (2023). A newly Synbiotic combination alleviates obesity by modulating the gut microbiota-fat Axis and inhibiting the hepatic TLR4/NF-κB signaling pathway. Mol. Nutr. Food Res. 67:e2300141. doi: 10.1002/mnfr.202300141, 37594720

[ref67] Kasahara N. Imi Y. Amano R. Shinohara M. Okada K. Hosokawa Y. . (2023). A gut microbial metabolite of linoleic acid ameliorates liver fibrosis by inhibiting TGF-β signaling in hepatic stellate cells. Sci. Rep. 13:18983. doi: 10.1038/s41598-023-46404-5 37923895 PMC10624680

[ref68] Kaye D. M. Shihata W. A. Jama H. A. Tsyganov K. Ziemann M. Kiriazis H. . (2020). Deficiency of prebiotic Fiber and insufficient signaling through gut metabolite-sensing receptors leads to cardiovascular disease. Circulation 141, 1393–1403. doi: 10.1161/CIRCULATIONAHA.119.043081, 32093510

[ref69] Ke X. Walker A. Haange S. B. Lagkouvardos I. Liu Y. Schmitt-Kopplin P. . (2019). Synbiotic-driven improvement of metabolic disturbances is associated with changes in the gut microbiome in diet-induced obese mice. Mol. Metab. 22, 96–109. doi: 10.1016/j.molmet.2019.01.012, 30792016 PMC6437638

[ref70] Kobyliak N. Falalyeyeva T. Bodnar P. Beregova T. (2017). Probiotics supplemented with Omega-3 fatty acids are more effective for hepatic steatosis reduction in an animal model of obesity. Probiotics Antimicrob. Proteins 9, 123–130. doi: 10.1007/s12602-016-9230-1 27660157

[ref71] Koduru L. Lakshmanan M. Hoon S. Lee D. Y. Lee Y. K. Ow D. S. (2022). Systems biology of gut microbiota-human receptor interactions: toward anti-inflammatory probiotics. Front. Microbiol. 13:846555. doi: 10.3389/fmicb.2022.846555, 35308387 PMC8928190

[ref72] Koeth R. A. Wang Z. Levison B. S. Buffa J. A. Org E. Sheehy B. T. . (2013). Intestinal microbiota metabolism of L-carnitine, a nutrient in red meat, promotes atherosclerosis. Nat. Med. 19, 576–585. doi: 10.1038/nm.3145, 23563705 PMC3650111

[ref73] Kondo S. Xiao J. Z. Satoh T. Odamaki T. Takahashi S. Sugahara H. . (2010). Antiobesity effects of *Bifidobacterium breve* strain B-3 supplementation in a mouse model with high-fat diet-induced obesity. Biosci. Biotechnol. Biochem. 74, 1656–1661. doi: 10.1271/bbb.100267 20699581

[ref74] Kootte R. S. Levin E. Salojärvi J. Smits L. P. Hartstra A. V. Udayappan S. D. . (2017). Improvement of insulin sensitivity after lean donor feces in metabolic syndrome is driven by baseline intestinal microbiota composition. Cell Metab. 26, 611–619.e616. doi: 10.1016/j.cmet.2017.09.008, 28978426

[ref75] Kumar S. S. Fathima A. Srihari P. Jamma T. (2024). Host-gut microbiota derived secondary metabolite mediated regulation of Wnt/β-catenin pathway: a potential therapeutic axis in IBD and CRC. Front. Oncol. 14:1392565. doi: 10.3389/fonc.2024.1392565 38706602 PMC11066261

[ref76] Kuzmich N. N. Sivak K. V. Chubarev V. N. Porozov Y. B. Savateeva-Lyubimova T. N. Peri F. (2017). TLR4 signaling pathway modulators as potential therapeutics in inflammation and Sepsis. Vaccines (Basel) 5:34. doi: 10.3390/vaccines5040034, 28976923 PMC5748601

[ref77] Lai X. Liu S. Miao J. Shen R. Wang Z. Zhang Z. . (2024). *Eubacterium siraeum* suppresses fat deposition via decreasing the tyrosine-mediated PI3K/AKT signaling pathway in high-fat diet-induced obesity. Microbiome 12:223. doi: 10.1186/s40168-024-01944-4 39478562 PMC11526712

[ref78] Lee C. H. Han Y. Ryu J. Y. Jung M. Park C. R. Jang M. R. . (2025). A novel strain *Bifidobacterium longum* subsp. longum HN001 ameliorates high-fat diet-induced obesity in mice through microbiome-associated short-chain fatty acids. Probiotics Antimicrob. Proteins. in press. doi: 10.1007/s12602-025-10766-1 PMC13176213 41085592

[ref79] Lee J. Park S. Oh N. Park J. Kwon M. Seo J. . (2021). Oral intake of *Lactobacillus plantarum* L-14 extract alleviates TLR2- and AMPK-mediated obesity-associated disorders in high-fat-diet-induced obese C57BL/6J mice. Cell Prolif. 54:e13039. doi: 10.1111/cpr.13039, 33830560 PMC8168423

[ref80] Lee N. R. Kwon T. J. Chung E. C. Bae J. Soung S. H. Tak H. J. . (2024). Combination of Lacticaseibacillus paracasei BEPC22 and Lactiplantibacillus plantarum BELP53 attenuates fat accumulation and alters the metabolome and gut microbiota in mice with high-fat diet-induced obesity. Food Funct. 15, 647–662. doi: 10.1039/D3FO03557C, 38099933

[ref81] Ley R. E. Hamady M. Lozupone C. Turnbaugh P. J. Ramey R. R. Bircher J. S. . (2008). Evolution of mammals and their gut microbes. Science 320, 1647–1651. doi: 10.1126/science.1155725, 18497261 PMC2649005

[ref82] Li A. Zhang J. Zhang X. Wang J. Wang S. Xiao X. . (2017). Angiotensin II induces connective tissue growth factor expression in human hepatic stellate cells by a transforming growth factor β-independent mechanism. Sci. Rep. 7:7841. doi: 10.1038/s41598-017-08334-x 28798388 PMC5552744

[ref83] Li D. Xu Z. Li Y. Gan L. Wu P. Wu R. . (2022). Polysaccharides from Callerya speciosa alleviate metabolic disorders and gut microbiota dysbiosis in diet-induced obese C57BL/6 mice. Food Funct. 13, 8662–8675. doi: 10.1039/D2FO00337F, 35904346

[ref84] Li G. H. Han F. F. Kalafatis E. Kong Q. P. Xiao W. (2025). Systems modeling reveals shared metabolic dysregulation and potential treatments in ME/CFS and long COVID. Int. J. Mol. Sci. 26:6082. doi: 10.3390/ijms26136082 40649860 PMC12250530

[ref85] Li M. Wang J. Wang F. Strappe P. Liu W. Zheng J. . (2021). Microbiota fermentation characteristics of acylated starches and the regulation mechanism of short-chain fatty acids on hepatic steatosis. Food Funct. 12, 8659–8668. doi: 10.1039/D1FO01226F, 34346457

[ref86] Li P. Wu Y. Bi R. Cao C. Hu J. Chen X. . (2025). Dietary *Enterococcus faecium* NCIMB 11181 supplementation mitigates intestinal and systemic inflammation induced by avian pathogenic *Escherichia coli* O78 infection in broiler chickens. Poult. Sci. 104:105656. doi: 10.1016/j.psj.2025.105656 40815990 PMC12362686

[ref87] Li X. Q. Zhu Y. H. Zhang H. F. Yue Y. Cai Z. X. Lu Q. P. . (2012). Risks associated with high-dose *Lactobacillus rhamnosus* in an *Escherichia coli* model of piglet diarrhoea: intestinal microbiota and immune imbalances. PLoS One 7:e40666. doi: 10.1371/journal.pone.0040666, 22848393 PMC3407149

[ref88] Li Y. C. Li Y. Liu L. Y. Chen Y. Zi T. Q. Du S. S. . (2015). The ratio of dietary branched-chain amino acids is associated with a lower prevalence of obesity in young northern Chinese adults: an internet-based cross-sectional study. Nutrients 7, 9573–9589. doi: 10.3390/nu7115486, 26593945 PMC4663614

[ref89] Liang L. Liu L. Zhou W. Yang C. Mai G. Li H. . (2022). Gut microbiota-derived butyrate regulates gut mucus barrier repair by activating the macrophage/WNT/ERK signaling pathway. Clin. Sci. (Lond.) 136, 291–307. doi: 10.1042/CS20210778, 35194640

[ref90] Lin X. Han H. Wang N. Wang C. Qi M. Wang J. . (2024). The gut microbial regulation of epigenetic modification from a metabolic perspective. Int. J. Mol. Sci. 25:7175. doi: 10.3390/ijms25137175, 39000282 PMC11241073

[ref91] Liu J. L. Xu X. Rixiati Y. Wang C. Y. Ni H. L. Chen W. S. . (2024). Dysfunctional circadian clock accelerates cancer metastasis by intestinal microbiota triggering accumulation of myeloid-derived suppressor cells. Cell Metab. 36, 1320–1334.e1329. doi: 10.1016/j.cmet.2024.04.019, 38838643

[ref92] Liu K. He X. Huang J. Yu S. Cui M. Gao M. . (2023). Short-chain fatty acid-butyric acid ameliorates granulosa cells inflammation through regulating METTL3-mediated N6-methyladenosine modification of FOSL2 in polycystic ovarian syndrome. Clin. Epigenetics 15:86. doi: 10.1186/s13148-023-01487-9 37179374 PMC10183145

[ref93] Liu Y. Yang J. Liu X. Liu R. Wang Y. Huang X. . (2023). Dietary folic acid addition reduces abdominal fat deposition mediated by alterations in gut microbiota and SCFA production in broilers. Anim. Nutr. 12, 54–62. doi: 10.1016/j.aninu.2022.08.013 36439290 PMC9684696

[ref94] Lu D. Tiezzi F. Schillebeeckx C. McNulty N. P. Schwab C. Shull C. . (2018). Host contributes to longitudinal diversity of fecal microbiota in swine selected for lean growth. Microbiome 6:4. doi: 10.1186/s40168-017-0384-1 29301569 PMC5755158

[ref95] Lu Y. Feng J. Yan Y. Qiu J. Feng L. (2025). LLTH induces white adipose tissue browning via NF κB inhibition in ATM. Sci. Rep. 15:37716. doi: 10.1038/s41598-025-21537-x 41152352 PMC12569381

[ref96] Lu Z. Zhang C. Zhang J. Su W. Wang G. Wang Z. (2025). The kynurenine pathway and indole pathway in tryptophan metabolism influence tumor progression. Cancer Med. 14:e70703. doi: 10.1002/cam4.70703 40103267 PMC11919716

[ref97] Luan C. Wang Y. Li J. Zhou N. Song G. Ni Z. . (2025). Branched-chain amino acid supplementation enhances substrate metabolism, exercise efficiency and reduces post-exercise fatigue in active young males. Nutrients 17:1290. doi: 10.3390/nu17071290, 40219047 PMC11990590

[ref99] Luo C. Yang Y. Xia L. Zhou K. Liu C. Yao L. . (2025). Multi-omics and experimental insights into the protective effects of Sesquiterpenoid lactones from Eupatorium lindleyanum DC. In acute lung injury: regulation of PI3K-Akt and MAPK-NF-κB pathways. Pharmaceuticals (Basel) 18:1523. doi: 10.3390/ph18101523 41155638 PMC12567390

[ref100] Luo P. Lednovich K. Xu K. Nnyamah C. Layden B. T. Xu P. (2022). Central and peripheral regulations mediated by short-chain fatty acids on energy homeostasis. Transl. Res. 248, 128–150. doi: 10.1016/j.trsl.2022.06.003, 35688319 PMC12553404

[ref101] Luu M. Pautz S. Kohl V. Singh R. Romero R. Lucas S. . (2019). The short-chain fatty acid pentanoate suppresses autoimmunity by modulating the metabolic-epigenetic crosstalk in lymphocytes. Nat. Commun. 10:760. doi: 10.1038/s41467-019-08711-2 30770822 PMC6377655

[ref102] Luu M. Riester Z. Baldrich A. Reichardt N. Yuille S. Busetti A. . (2021). Microbial short-chain fatty acids modulate CD8(+) T cell responses and improve adoptive immunotherapy for cancer. Nat. Commun. 12:4077. doi: 10.1038/s41467-021-24331-1 34210970 PMC8249424

[ref103] Lyu W. Liu X. Lu L. Dai B. Wang W. Yang H. . (2021). Cecal microbiota modulates fat deposition in Muscovy ducks. Front. Vet. Sci. 8:609348. doi: 10.3389/fvets.2021.609348, 33869315 PMC8044358

[ref104] Ma L. Tao S. Song T. Lyu W. Li Y. Wang W. . (2024). Clostridium butyricum and carbohydrate active enzymes contribute to the reduced fat deposition in pigs. iMeta 3:e160. doi: 10.1002/imt2.160, 38868506 PMC10989082

[ref105] Ma Y. Zhong Y. Tang W. Valencak T. G. Liu J. Deng Z. . (2025). *Lactobacillus reuteri* ZJ617 attenuates metabolic syndrome via microbiota-derived spermidine. Nat. Commun. 16:877. doi: 10.1038/s41467-025-56105-4 39837844 PMC11750987

[ref106] Madhu B. Miller B. M. Levy M. (2023). Single-cell analysis and spatial resolution of the gut microbiome. Front. Cell. Infect. Microbiol. 13:1271092. doi: 10.3389/fcimb.2023.1271092 37860069 PMC10582963

[ref107] Maffei M. E. (2020). 5-Hydroxytryptophan (5-HTP): natural occurrence, analysis, biosynthesis, biotechnology, physiology and toxicology. Int. J. Mol. Sci. 22:181. doi: 10.3390/ijms22010181 33375373 PMC7796270

[ref108] Maj M. A. Burrin D. G. Manjarín R. (2023). Decreased FXR Agonism in the bile acid Pool is associated with impaired FXR signaling in a pig model of pediatric NAFLD. Biomedicine 11:3303. doi: 10.3390/biomedicines11123303, 38137523 PMC10740974

[ref109] Majumdar A. Siva Venkatesh I. P. Swarup V. Basu A. (2024). Short-chain fatty acids abrogate Japanese encephalitis virus-induced inflammation in microglial cells via miR-200a-3p/ZBTB20/IKβα axis. MBio 15:e0132124. doi: 10.1128/mbio.01321-24, 38869276 PMC11253640

[ref110] Mamun M. A. A. Rakib A. Mandal M. Singh U. P. (2025). Impact of a high-fat diet on the gut microbiome: a comprehensive study of microbial and metabolite shifts during obesity. Cells 14:463. doi: 10.3390/cells14060463, 40136712 PMC11940932

[ref111] Mandaliya D. K. Patel S. Seshadri S. (2021). The combinatorial effect of acetate and propionate on high-fat diet induced diabetic inflammation or Metaflammation and T cell polarization. Inflammation 44, 68–79. doi: 10.1007/s10753-020-01309-7, 32978698

[ref112] Martinez-Guryn K. Hubert N. Frazier K. Urlass S. Musch M. W. Ojeda P. . (2018). Small intestine microbiota regulate host digestive and absorptive adaptive responses to dietary lipids. Cell Host Microbe 23, 458–469.e455. doi: 10.1016/j.chom.2018.03.011, 29649441 PMC5912695

[ref113] Martinho D. V. Nobari H. Faria A. Field A. Duarte D. Sarmento H. (2022). Oral branched-chain amino acids supplementation in athletes: a systematic review. Nutrients 14:4002. doi: 10.3390/nu14194002, 36235655 PMC9571679

[ref114] May K. S. den Hartigh L. J. (2023). Gut microbial-derived short chain fatty acids: impact on adipose tissue physiology. Nutrients 15:272. doi: 10.3390/nu15020272, 36678142 PMC9865590

[ref115] Meier K. H. U. Trouillon J. Li H. Lang M. Fuhrer T. Zamboni N. . (2023). Metabolic landscape of the male mouse gut identifies different niches determined by microbial activities. Nat. Metab. 5, 968–980. doi: 10.1038/s42255-023-00802-1, 37217759 PMC10290957

[ref116] Miao X. Alidadipour A. Saed V. Sayyadi F. Jadidi Y. Davoudi M. . (2024). Hepatokines: unveiling the molecular and cellular mechanisms connecting hepatic tissue to insulin resistance and inflammation. Acta Diabetol. 61, 1339–1361. doi: 10.1007/s00592-024-02335-9, 39031190

[ref117] Million M. Maraninchi M. Henry M. Armougom F. Richet H. Carrieri P. . (2012). Obesity-associated gut microbiota is enriched in Lactobacillus reuteri and depleted in Bifidobacterium animalis and *Methanobrevibacter smithii*. Int. J. Obes. 36, 817–825. doi: 10.1038/ijo.2011.153, 21829158 PMC3374072

[ref118] Młynarska E. Bojdo K. Bulicz A. Frankenstein H. Gąsior M. Kustosik N. . (2025). Obesity as a multifactorial chronic disease: molecular mechanisms, systemic impact, and emerging digital interventions. Curr. Issues Mol. Biol. 47:787. doi: 10.3390/cimb47100787, 41150735 PMC12564886

[ref119] Moreno-Indias I. Cardona F. Tinahones F. J. Queipo-Ortuño M. I. (2014). Impact of the gut microbiota on the development of obesity and type 2 diabetes mellitus. Front. Microbiol. 5:190. doi: 10.3389/fmicb.2014.00190, 24808896 PMC4010744

[ref120] Mouries J. Brescia P. Silvestri A. Spadoni I. Sorribas M. Wiest R. . (2019). Microbiota-driven gut vascular barrier disruption is a prerequisite for non-alcoholic steatohepatitis development. J. Hepatol. 71, 1216–1228. doi: 10.1016/j.jhep.2019.08.005 31419514 PMC6880766

[ref121] Mu J. Tan F. Zhou X. Zhao X. (2020). *Lactobacillus fermentum* CQPC06 in naturally fermented pickles prevents non-alcoholic fatty liver disease by stabilizing the gut-liver axis in mice. Food Funct. 11, 8707–8723. doi: 10.1039/D0FO01823F, 32945305

[ref122] Mukhopadhya I. Louis P. (2025). Gut microbiota-derived short-chain fatty acids and their role in human health and disease. Nat. Rev. Microbiol. 23, 635–651. doi: 10.1038/s41579-025-01183-w, 40360779

[ref123] Natividad J. M. Agus A. Planchais J. Lamas B. Jarry A. C. Martin R. . (2018). Impaired aryl hydrocarbon receptor ligand production by the gut microbiota is a key factor in metabolic syndrome. Cell Metab. 28, 737–749.e734. doi: 10.1016/j.cmet.2018.07.001, 30057068

[ref124] Nohesara S. Mostafavi Abdolmaleky H. Pirani A. Pettinato G. Thiagalingam S. (2025). The obesity-epigenetics-microbiome Axis: strategies for therapeutic intervention. Nutrients 17:1564. doi: 10.3390/nu17091564 40362873 PMC12073275

[ref125] Nshanian M. Gruber J. J. Geller B. S. Chleilat F. Lancaster S. M. White S. M. . (2025). Short-chain fatty acid metabolites propionate and butyrate are unique epigenetic regulatory elements linking diet, metabolism and gene expression. Nat. Metab. 7, 196–211. doi: 10.1038/s42255-024-01191-9, 39789354 PMC11774759

[ref126] Pandey S. P. Bender M. J. McPherson A. C. Phelps C. M. Sanchez L. M. Rana M. . (2022). Tet2 deficiency drives liver microbiome dysbiosis triggering Tc1 cell autoimmune hepatitis. Cell Host Microbe 30, 1003–1019.e1010. doi: 10.1016/j.chom.2022.05.006, 35658976 PMC9841318

[ref127] Panichsillaphakit E. Chongpison Y. Saengpanit P. Kwanbunbumpen T. Uaariyapanichkul J. Chomtho S. . (2021). Children's eating behavior questionnaire correlated with body compositions of Thai children and adolescents with obesity: a pilot study. J. Nutr. Metab. 2021:6496134. doi: 10.1155/2021/6496134, 33510908 PMC7822704

[ref128] Paone P. Cani P. D. (2020). Mucus barrier, mucins and gut microbiota: the expected slimy partners? Gut 69, 2232–2243. doi: 10.1136/gutjnl-2020-322260, 32917747 PMC7677487

[ref129] Pappo I. Becovier H. Berry E. M. Freund H. R. (1991). Polymyxin B reduces cecal flora, TNF production and hepatic steatosis during total parenteral nutrition in the rat. J. Surg. Res. 51, 106–112. doi: 10.1016/0022-4804(91)90078-Z, 1907698

[ref130] Patil A. Singh N. Patwekar M. Patwekar F. Patil A. Gupta J. K. . (2025). AI-driven insights into the microbiota: figuring out the mysterious world of the gut. Intell. Pharm. 3, 46–52. doi: 10.1016/j.ipha.2024.08.003

[ref131] Pearson J. A. Ding H. Hu C. Peng J. Galuppo B. Wong F. S. . (2022). IgM-associated gut bacteria in obesity and type 2 diabetes in C57BL/6 mice and humans. Diabetologia 65, 1398–1411. doi: 10.1007/s00125-022-05711-8, 35587276 PMC9283171

[ref132] Pedersen S. S. Ingerslev L. R. Olsen M. Prause M. Billestrup N. (2024). Butyrate functions as a histone deacetylase inhibitor to protect pancreatic beta cells from IL-1β-induced dysfunction. FEBS J. 291, 566–583. doi: 10.1111/febs.17005, 37985375

[ref133] Qin L. Q. Xun P. Bujnowski D. Daviglus M. L. Van Horn L. Stamler J. . (2011). Higher branched-chain amino acid intake is associated with a lower prevalence of being overweight or obese in middle-aged east Asian and Western adults. J. Nutr. 141, 249–254. doi: 10.3945/jn.110.128520, 21169225 PMC3021443

[ref134] Quan Y. Yin Z. Chen S. Lang J. Han L. Yi J. . (2022). The gut-lung axis: gut microbiota changes associated with pulmonary fibrosis in mouse models induced by bleomycin. Front. Pharmacol. 13:985223. doi: 10.3389/fphar.2022.985223, 36249808 PMC9561135

[ref135] Rajani C. Jia W. (2018). Disruptions in gut microbial-host co-metabolism and the development of metabolic disorders. Clin. Sci. (Lond.) 132, 791–811. doi: 10.1042/CS20171328, 29661926

[ref136] Ramasinghe C. Bordiga M. Xu B. (2025). A comprehensive review of the triangular relationship among diet, gut microbiota, and aging. Int. J. Mol. Sci. 26:8785. doi: 10.3390/ijms26188785, 41009354 PMC12469625

[ref137] Remely M. Aumueller E. Merold C. Dworzak S. Hippe B. Zanner J. . (2014). Effects of short chain fatty acid producing bacteria on epigenetic regulation of FFAR3 in type 2 diabetes and obesity. Gene 537, 85–92. doi: 10.1016/j.gene.2013.11.081, 24325907

[ref138] Ridaura V. K. Faith J. J. Rey F. E. Cheng J. Duncan A. E. Kau A. L. . (2013). Gut microbiota from twins discordant for obesity modulate metabolism in mice. Science 341:1241214. doi: 10.1126/science.1241214 24009397 PMC3829625

[ref139] Ridlon J. M. Harris S. C. Bhowmik S. Kang D. J. Hylemon P. B. (2016). Consequences of bile salt biotransformations by intestinal bacteria. Gut Microbes 7, 22–39. doi: 10.1080/19490976.2015.1127483, 26939849 PMC4856454

[ref140] Rimal B. Collins S. L. Tanes C. E. Rocha E. R. Granda M. A. Solanki S. . (2024). Bile salt hydrolase catalyses formation of amine-conjugated bile acids. Nature 626, 859–863. doi: 10.1038/s41586-023-06990-w, 38326609 PMC10881385

[ref141] Rodriguez D. M. Benninghoff A. D. Aardema N. D. J. Phatak S. Hintze K. J. (2019). Basal diet determined long-term composition of the gut microbiome and mouse phenotype to a greater extent than fecal microbiome transfer from lean or obese human donors. Nutrients 11:1630. doi: 10.3390/nu11071630, 31319545 PMC6682898

[ref142] Rojas I. Y. Moyer B. J. Ringelberg C. S. Wilkins O. M. Pooler D. B. Ness D. B. . (2021). Kynurenine-induced aryl hydrocarbon receptor signaling in mice causes body mass gain, liver steatosis, and hyperglycemia. Obesity (Silver Spring) 29, 337–349. doi: 10.1002/oby.23065 33491319 PMC10782555

[ref143] Rouskas K. Guela M. Pantoura M. Pagkalos I. Hassapidou M. Lalama E. . (2025). The influence of an AI-driven personalized nutrition program on the human gut microbiome and its health implications. Nutrients 17:1260. doi: 10.3390/nu17071260, 40219016 PMC11990151

[ref144] Ryu S. Kyoung H. Park K. I. Oh S. Song M. Kim Y. (2022). Postbiotic heat-killed lactobacilli modulates on body weight associated with gut microbiota in a pig model. AMB Express 12:83. doi: 10.1186/s13568-022-01424-8 35767074 PMC9243212

[ref145] Schugar R. C. Shih D. M. Warrier M. Helsley R. N. Burrows A. Ferguson D. . (2017). The TMAO-producing enzyme Flavin-containing monooxygenase 3 regulates obesity and the Beiging of White adipose tissue. Cell Rep. 19, 2451–2461. doi: 10.1016/j.celrep.2017.05.077, 28636934 PMC5672822

[ref146] Shan Z. Sun T. Huang H. Chen S. Chen L. Luo C. . (2017). Association between microbiota-dependent metabolite trimethylamine-N-oxide and type 2 diabetes. Am. J. Clin. Nutr. 106, 888–894. doi: 10.3945/ajcn.117.157107, 28724646

[ref147] Shanmugham M. Bellanger S. Leo C. H. (2023). Gut-derived metabolite, trimethylamine-N-oxide (TMAO) in cardio-metabolic diseases: detection, mechanism, and potential therapeutics. Pharmaceuticals (Basel) 16:504. doi: 10.3390/ph16040504 37111261 PMC10142468

[ref148] Sharma S. A. Oladejo S. O. Kuang Z. (2025). Chemical interplay between gut microbiota and epigenetics: implications in circadian biology. Cell Chem. Biol. 32, 61–82. doi: 10.1016/j.chembiol.2024.04.016, 38776923 PMC11569273

[ref149] ShiR. WeiJ. YeJ. SongX. YangX. ZhangY. . (2025). The novel synbiotic (Lactiplantibacillus plantarum and galacto-oligosaccharides) ameliorates obesity-related metabolic dysfunction: arginine as a key mediator signaling molecule. J. Adv. Res. in press. doi: 10.1016/j.jare.2025.06.041, 40545235

[ref150] Shimizu H. Masujima Y. Ushiroda C. Mizushima R. Taira S. Ohue-Kitano R. . (2019). Dietary short-chain fatty acid intake improves the hepatic metabolic condition via FFAR3. Sci. Rep. 9:16574. doi: 10.1038/s41598-019-53242-x 31719611 PMC6851370

[ref151] Soundharrajan I. Kuppusamy P. Srisesharam S. Lee J. C. Sivanesan R. Kim D. . (2020). Positive metabolic effects of selected probiotic bacteria on diet-induced obesity in mice are associated with improvement of dysbiotic gut microbiota. FASEB J. 34, 12289–12307. doi: 10.1096/fj.202000971R, 32701200

[ref152] Spillane M. Emerson C. Willoughby D. S. (2013). The effects of 8 weeks of heavy resistance training and branched-chain amino acid supplementation on body composition and muscle performance. J. Int. Soc. Sports Nutr. 10:P25. doi: 10.1186/1550-2783-10-S1-P25 24620007

[ref153] Srivastava S. Mohanty B. (2025). Probiotics as an adjunct ameliorates ovarian toxicity in Endotoxemic mice via modulating TLR 4/MyD88/NF-κB Signalling pathway: insights from in vivo and in silico study. Reprod. Sci. in press. doi: 10.1007/s43032-025-02009-z, 41145945

[ref154] Stoppani J. Scheett T. Pena J. Rudolph C. Charlebois D. (2009). 2009 international society of sports nutrition conference and expo new orleans, la, USA. 14–15 june 2009. Abstracts. J. Int. Soc. Sports Nutr. 6, P1–p19. doi: 10.1186/1550-2783-6-s1-p1 19660093 PMC3313152

[ref155] Streck E. L. Bussular F. P. Wessler L. B. Duarte M. B. Rezende V. L. Rodrigues M. S. . (2021). Administration of branched-chain amino acids alters epigenetic regulatory enzymes in an animal model of maple syrup urine disease. Metab. Brain Dis. 36, 247–254. doi: 10.1007/s11011-020-00631-1, 33098071

[ref156] Sun C. Mao S. Chen S. Zhang W. Liu C. (2021). PPARs-orchestrated metabolic homeostasis in the adipose tissue. Int. J. Mol. Sci. 22:8974. doi: 10.3390/ijms22168974, 34445679 PMC8396609

[ref157] Suriano F. Vieira-Silva S. Falony G. Roumain M. Paquot A. Pelicaen R. . (2021). Novel insights into the genetically obese (Ob/Ob) and diabetic (db/db) mice: two sides of the same coin. Microbiome 9:147. doi: 10.1186/s40168-021-01097-8 34183063 PMC8240277

[ref158] Swanson K. S. Allenspach K. Amos G. Auchtung T. A. Bassett S. A. Bjørnvad C. R. . (2025). Use of biotics in animals: impact on nutrition, health, and food production. J. Anim. Sci. 103:skaf061. doi: 10.1093/jas/skaf061, 40036559 PMC12010704

[ref159] Takeuchi T. Kameyama K. Miyauchi E. Nakanishi Y. Kanaya T. Fujii T. . (2023). Fatty acid overproduction by gut commensal microbiota exacerbates obesity. Cell Metab. 35, 361–375.e369. doi: 10.1016/j.cmet.2022.12.013, 36652945

[ref160] Tarallo S. Ferrero G. De Filippis F. Francavilla A. Pasolli E. Panero V. . (2022). Stool microRNA profiles reflect different dietary and gut microbiome patterns in healthy individuals. Gut 71, 1302–1314. doi: 10.1136/gutjnl-2021-325168, 34315772 PMC9185830

[ref161] Taubenheim J. Kadibalban A. S. Zimmermann J. Taubenheim C. Tran F. Schreiber S. . (2025). Metabolic modeling reveals a multi-level deregulation of host-microbiome metabolic networks in IBD. Nat. Commun. 16:5120. doi: 10.1038/s41467-025-60233-2 40456745 PMC12130198

[ref162] Than A. Cheng Y. Foh L. C. Leow M. K. Lim S. C. Chuah Y. J. . (2012). Apelin inhibits adipogenesis and lipolysis through distinct molecular pathways. Mol. Cell. Endocrinol. 362, 227–241. doi: 10.1016/j.mce.2012.07.002, 22842084

[ref163] Thomas S. P. Denu J. M. (2021). Short-chain fatty acids activate acetyltransferase p300. eLife 10:e72171. doi: 10.7554/eLife.72171, 34677127 PMC8585482

[ref164] Tsukamoto S. Suzuki T. Wakui H. Uehara T. Ichikawa J. Okuda H. . (2023). Angiotensin II type 1 receptor-associated protein in immune cells: a possible key factor in the pathogenesis of visceral obesity. Metabolism 149:155706. doi: 10.1016/j.metabol.2023.155706, 37856903

[ref165] Tu J. Wang Y. Jin L. Huang W. (2022). Bile acids, gut microbiota and metabolic surgery. Front. Endocrinol. (Lausanne) 13:929530. doi: 10.3389/fendo.2022.929530, 36072923 PMC9441571

[ref166] Tung Y. C. Chou R. F. Nagabhushanam K. Ho C. T. Pan M. H. (2020). 3'-Hydroxydaidzein improves obesity through the induced Browning of beige adipose and modulation of gut microbiota in mice with obesity induced by a high-fat diet. J. Agric. Food Chem. 68, 14513–14522. doi: 10.1021/acs.jafc.0c06138, 33231468

[ref167] Tung Y. C. Shih Y. A. Nagabhushanam K. Ho C. T. Cheng A. C. Pan M. H. (2021). Coleus forskohlii and *Garcinia indica* extracts attenuated lipid accumulation by regulating energy metabolism and modulating gut microbiota in obese mice. Food Res. Int. 142:110143. doi: 10.1016/j.foodres.2021.110143, 33773654

[ref168] Turnbaugh P. J. Ley R. E. Mahowald M. A. Magrini V. Mardis E. R. Gordon J. I. (2006). An obesity-associated gut microbiome with increased capacity for energy harvest. Nature 444, 1027–1031. doi: 10.1038/nature05414, 17183312

[ref169] Tyagi A. M. Yu M. Darby T. M. Vaccaro C. Li J. Y. Owens J. A. . (2018). The microbial metabolite butyrate stimulates bone formation via T regulatory cell-mediated regulation of WNT10B expression. Immunity 49, 1116–1131.e1117. doi: 10.1016/j.immuni.2018.10.013, 30446387 PMC6345170

[ref170] Urmi J. F. Itoh H. Muramatsu-Kato K. Kohmura-Kobayashi Y. Hariya N. Jain D. . (2019). Plasticity of histone modifications around Cidea and Cidec genes with secondary bile in the amelioration of developmentally-programmed hepatic steatosis. Sci. Rep. 9:17100. doi: 10.1038/s41598-019-52943-7 31745102 PMC6863835

[ref171] Vallianou N. Stratigou T. Christodoulatos G. S. Tsigalou C. Dalamaga M. (2020). Probiotics, prebiotics, Synbiotics, Postbiotics, and obesity: current evidence, controversies, and perspectives. Curr. Obes. Rep. 9, 179–192. doi: 10.1007/s13679-020-00379-w, 32472285

[ref172] Velazquez-Villegas L. A. Perino A. Lemos V. Zietak M. Nomura M. Pols T. W. H. . (2018). TGR5 signalling promotes mitochondrial fission and beige remodelling of white adipose tissue. Nat. Commun. 9:245. doi: 10.1038/s41467-017-02068-0 29339725 PMC5770450

[ref173] Voronova V. Sokolov V. Al-Khaifi A. Straniero S. Kumar C. Peskov K. . (2020). A physiology-based model of bile acid distribution and metabolism under healthy and pathologic conditions in human beings. Cell. Mol. Gastroenterol. Hepatol. 10, 149–170. doi: 10.1016/j.jcmgh.2020.02.005, 32112828 PMC7240226

[ref174] Wang C. Zheng K. Wang D. Yu H. Zhao Y. Fang H. . (2024). Effects of adding bile acids to dietary storage japonica brown rice on growth performance, meat quality, and intestinal microbiota of growing-finishing min pigs. Front. Vet. Sci. 11:1349754. doi: 10.3389/fvets.2024.1349754 38711539 PMC11070551

[ref175] Wang K. Chen G. Cao G. Xu Y. Wang Y. Yang C. (2019). Effects of Clostridium butyricum and *Enterococcus faecalis* on growth performance, intestinal structure, and inflammation in lipopolysaccharide-challenged weaned piglets. J. Anim. Sci. 97, 4140–4151. doi: 10.1093/jas/skz235, 31310662 PMC6776315

[ref176] Wang L. Feng J. Deng Y. Yang Q. Wei Q. Ye D. . (2022). CCAAT/enhancer-binding proteins in fibrosis: complex roles beyond conventional understanding. Research (Wash D C) 2022:9891689. doi: 10.34133/2022/9891689, 36299447 PMC9575473

[ref177] Wang N. Li C. Zhang Z. (2024). Arctigenin ameliorates high-fat diet-induced metabolic disorders by reshaping gut microbiota and modulating GPR/HDAC3 and TLR4/NF-κB pathways. Phytomedicine 135:156123. doi: 10.1016/j.phymed.2024.156123 39396403

[ref178] Wang R. Halimulati M. Huang X. Ma Y. Li L. Zhang Z. (2023). Sulforaphane-driven reprogramming of gut microbiome and metabolome ameliorates the progression of hyperuricemia. J. Adv. Res. 52, 19–28. doi: 10.1016/j.jare.2022.11.003, 36371056 PMC10555773

[ref179] Wise J. L. Cummings B. P. (2022). The 7-α-dehydroxylation pathway: an integral component of gut bacterial bile acid metabolism and potential therapeutic target. Front. Microbiol. 13:1093420. doi: 10.3389/fmicb.2022.1093420, 36699589 PMC9868651

[ref180] Wu S. C. Lo Y. M. Lee J. H. Chen C. Y. Chen T. W. Liu H. W. . (2022). Stomatin modulates adipogenesis through the ERK pathway and regulates fatty acid uptake and lipid droplet growth. Nat. Commun. 13:4174. doi: 10.1038/s41467-022-31825-z 35854007 PMC9296665

[ref181] Wu Y. L. Lin Z. J. Li C. C. Lin X. Shan S. K. Guo B. . (2023). Epigenetic regulation in metabolic diseases: mechanisms and advances in clinical study. Signal Transduct. Target. Ther. 8:98. doi: 10.1038/s41392-023-01333-7 36864020 PMC9981733

[ref182] Xue C. Li G. Zheng Q. Gu X. Shi Q. Su Y. . (2023). Tryptophan metabolism in health and disease. Cell Metab. 35, 1304–1326. doi: 10.1016/j.cmet.2023.06.004, 37352864

[ref183] Yan H. Diao H. Xiao Y. Li W. Yu B. He J. . (2016). Gut microbiota can transfer fiber characteristics and lipid metabolic profiles of skeletal muscle from pigs to germ-free mice. Sci. Rep. 6:31786. doi: 10.1038/srep31786 27545196 PMC4992887

[ref184] Yang H. Xiang Y. Robinson K. Wang J. Zhang G. Zhao J. . (2018). Gut microbiota is a major contributor to adiposity in pigs. Front. Microbiol. 9:3045. doi: 10.3389/fmicb.2018.03045 30619136 PMC6296290

[ref185] Yang J. Li G. Wang S. He M. Dong S. Wang T. . (2025). Butyrate prevents obesity accompanied by HDAC9-mediated Browning of White adipose tissue. Biomedicine 13:260. doi: 10.3390/biomedicines13020260 PMC11852213 40002674

[ref186] Yang L. Huang Y. Chen M. Ma X. Yu X. Ren D. . (2025). Pasteurized *Akkermansia muciniphila* AKK PROBIO attenuates obesity through gut microbiota-SCFA-GLP-1 Axis and potential involvement of AMPK/PPAR-α pathway. Probiotics Antimicrob. Proteins. in press. doi: 10.1007/s12602-025-10805-x 41123834

[ref187] Yang M. Q. Wang Z. J. Zhai C. B. Chen L. Q. (2024). Research progress on the application of 16S rRNA gene sequencing and machine learning in forensic microbiome individual identification. Front. Microbiol. 15:1360457. doi: 10.3389/fmicb.2024.1360457 38371926 PMC10869621

[ref188] Yang W. Yu T. Huang X. Bilotta A. J. Xu L. Lu Y. . (2020). Intestinal microbiota-derived short-chain fatty acids regulation of immune cell IL-22 production and gut immunity. Nat. Commun. 11:4457. doi: 10.1038/s41467-020-18262-6 32901017 PMC7478978

[ref189] Yang Y. Liu R. Sun Y. Wu B. He B. Jia Y. . (2024). Schisandrin B restores M1/M2 balance through miR-124 in lipopolysaccharide-induced BV2 cells. J. Pharm. Pharmacol. 76, 1352–1361. doi: 10.1093/jpp/rgae079 39024474

[ref190] Yang Z. He M. Austin J. Sayed D. Abdellatif M. (2023). Reducing branched-chain amino acids improves cardiac stress response in mice by decreasing histone H3K23 propionylation. J. Clin. Invest. 133:e169399. doi: 10.1172/JCI169399, 37669116 PMC10645387

[ref191] Yang Z. Huang S. Zou D. Dong D. He X. Liu N. . (2016). Metabolic shifts and structural changes in the gut microbiota upon branched-chain amino acid supplementation in middle-aged mice. Amino Acids 48, 2731–2745. doi: 10.1007/s00726-016-2308-y, 27539648

[ref192] Yao F. Jia R. Huang H. Yu Y. Mei L. Bai L. . (2019). Effect of *Lactobacillus paracasei* N1115 and fructooligosaccharides in nonalcoholic fatty liver disease. Arch. Med. Sci. 15, 1336–1344. doi: 10.5114/aoms.2019.86611, 31572482 PMC6764303

[ref193] Ye J. Lv L. Wu W. Li Y. Shi D. Fang D. . (2018). Butyrate protects mice against methionine-choline-deficient diet-induced non-alcoholic steatohepatitis by improving gut barrier function, attenuating inflammation and reducing endotoxin levels. Front. Microbiol. 9:1967. doi: 10.3389/fmicb.2018.01967 30186272 PMC6111843

[ref194] Yoneshiro T. Wang Q. Tajima K. Matsushita M. Maki H. Igarashi K. . (2019). BCAA catabolism in brown fat controls energy homeostasis through SLC25A44. Nature 572, 614–619. doi: 10.1038/s41586-019-1503-x, 31435015 PMC6715529

[ref195] Yoshida H. Ishii M. Akagawa M. (2019). Propionate suppresses hepatic gluconeogenesis via GPR43/AMPK signaling pathway. Arch. Biochem. Biophys. 672:108057. doi: 10.1016/j.abb.2019.07.022, 31356781

[ref196] Yu H. Li R. Huang H. Yao R. Shen S. (2018). Short-chain fatty acids enhance the lipid accumulation of 3T3-L1 cells by modulating the expression of enzymes of fatty acid metabolism. Lipids 53, 77–84. doi: 10.1002/lipd.12005, 29488641

[ref197] Yuan H. Wu X. Wu Q. Chatoff A. Megill E. Gao J. . (2023). Lysine catabolism reprograms tumour immunity through histone crotonylation. Nature 617, 818–826. doi: 10.1038/s41586-023-06061-0, 37198486 PMC11089809

[ref198] Zha A. Li W. Wang J. Bai P. Qi M. Liao P. . (2024). Trimethylamine oxide supplementation differentially regulates fat deposition in liver, longissimus dorsi muscle and adipose tissue of growing-finishing pigs. Anim. Nutr. 17, 25–35. doi: 10.1016/j.aninu.2023.12.006, 38464952 PMC10920132

[ref199] Zhang D. Jian Y. P. Zhang Y. N. Li Y. Gu L. T. Sun H. H. . (2023). Short-chain fatty acids in diseases. Cell Commun. Signal 21:212. doi: 10.1186/s12964-023-01219-9 37596634 PMC10436623

[ref200] Zhang H. Zhang W. Yun D. Li L. Zhao W. Li Y. . (2020). Alternate-day fasting alleviates diabetes-induced glycolipid metabolism disorders: roles of FGF21 and bile acids. J. Nutr. Biochem. 83:108403. doi: 10.1016/j.jnutbio.2020.108403, 32497958

[ref201] Zhang J. Sun J. Chen X. Nie C. Zhao J. Guan W. . (2018). Combination of Clostridium butyricum and corn bran optimized intestinal microbial fermentation using a weaned pig model. Front. Microbiol. 9:3091. doi: 10.3389/fmicb.2018.03091 30619170 PMC6305284

[ref202] Zhang R. Nakao T. Luo J. Xue Y. Cornuet P. Oertel M. . (2019). Activation of WNT/Beta-catenin signaling and regulation of the Farnesoid X receptor/Beta-catenin complex after murine bile duct ligation. Hepatol. Commun. 3, 1642–1655. doi: 10.1002/hep4.1430, 31832572 PMC6887668

[ref203] Zhang X. Gao X. Liu Z. Shao F. Yu D. Zhao M. . (2024). Microbiota regulates the TET1-mediated DNA hydroxymethylation program in innate lymphoid cell differentiation. Nat. Commun. 15:4792. doi: 10.1038/s41467-024-48794-0 38839760 PMC11153590

[ref204] Zhang Y. Jiang D. Jin Y. Jia H. Yang Y. Kim I. H. . (2021). Glycine attenuates *Citrobacter rodentium*-induced colitis by regulating ATF6-mediated endoplasmic reticulum stress in mice. Mol. Nutr. Food Res. 65:e2001065. doi: 10.1002/mnfr.202001065, 34075695

[ref205] Zhao Y. Wu J. Liu X. Chen X. Wang J. (2024). Decoding nature: multi-target anti-inflammatory mechanisms of natural products in the TLR4/NF-κB pathway. Front. Pharmacol. 15:1467193. doi: 10.3389/fphar.2024.1467193, 39877388 PMC11772364

[ref206] Zheng L. Zhan Y. Wang C. Fan Q. Sun D. Li Y. . (2024). Technological advances and challenges in constructing complex gut organoid systems. Front. Cell Dev. Biol. 12:1432744. doi: 10.3389/fcell.2024.1432744 39206092 PMC11349554

[ref207] Zheng M. Chao X. Zheng Y. Hong T. Wu W. Zhu Y. . (2024). A polysaccharide from edible red seaweed *Bangia fusco-purpurea* prevents obesity in high-fat diet-induced C57BL/6 mice. Int. J. Biol. Macromol. 283:137545. doi: 10.1016/j.ijbiomac.2024.137545 39542298

[ref208] Zheng W. Liu M. Lv X. He C. Yin J. Ma J. (2025). AhR governs lipid metabolism: the role of gut microbiota. Front. Microbiol. 16:1442282. doi: 10.3389/fmicb.2025.1442282 39944639 PMC11817270

[ref209] Zheng W. Tang S. Ren X. Song S. Ai C. (2024). Fucoidan alleviated colitis aggravated by fiber deficiency through protecting the gut barrier, suppressing the MAPK/NF-κB pathway, and modulating gut microbiota and metabolites. Front. Nutr. 11:1462584. doi: 10.3389/fnut.2024.1462584 39925971 PMC11802440

[ref210] Zheng X. Chen T. Jiang R. Zhao A. Wu Q. Kuang J. . (2021). Hyocholic acid species improve glucose homeostasis through a distinct TGR5 and FXR signaling mechanism. Cell Metab. 33, 791–803.e797. doi: 10.1016/j.cmet.2020.11.017, 33338411

[ref211] Zheng X. Huang W. Li Q. Chen Y. Wu L. Dong Y. . (2023). Membrane protein Amuc_1100 derived from *Akkermansia muciniphila* facilitates lipolysis and Browning via activating the AC3/PKA/HSL pathway. Microbiol. Spectr. 11:e0432322. doi: 10.1128/spectrum.04323-22, 36847500 PMC10100790

[ref212] Zhernakova D. V. Wang D. Liu L. Andreu-Sánchez S. Zhang Y. Ruiz-Moreno A. J. . (2024). Host genetic regulation of human gut microbial structural variation. Nature 625, 813–821. doi: 10.1038/s41586-023-06893-w, 38172637 PMC10808065

[ref213] Zhu Q. Song M. Azad M. A. K. Ma C. Yin Y. Kong X. (2022). Probiotics and Synbiotics addition to Bama Mini-pigs' diet improve carcass traits and meat quality by altering plasma metabolites and related gene expression of offspring. Front. Vet. Sci. 9:779745. doi: 10.3389/fvets.2022.779745, 35873696 PMC9301501

[ref214] Zhu X. Li K. Liu G. Wu R. Zhang Y. Wang S. . (2023). Microbial metabolite butyrate promotes anti-PD-1 antitumor efficacy by modulating T cell receptor signaling of cytotoxic CD8 T cell. Gut Microbes 15:2249143. doi: 10.1080/19490976.2023.2249143 37635362 PMC10464552

[ref215] Zsálig D. Berta A. Tóth V. Szabó Z. Simon K. Figler M. . (2023). A review of the relationship between gut microbiome and obesity. Appl. Sci. 13:610. doi: 10.3390/app13010610

